# Clonal relations in the mouse brain revealed by single-cell and spatial transcriptomics

**DOI:** 10.1038/s41593-022-01011-x

**Published:** 2022-02-24

**Authors:** Michael Ratz, Leonie von Berlin, Ludvig Larsson, Marcel Martin, Jakub Orzechowski Westholm, Gioele La Manno, Joakim Lundeberg, Jonas Frisén

**Affiliations:** 1 Department of Cell and Molecular Biology, Karolinska Institute, Stockholm, Sweden; 2 Science for Life Laboratory, KTH Royal Institute of Technology, Stockholm, Sweden; 3 Department of Biochemistry and Biophysics, National Bioinformatics Infrastructure Sweden, Science for Life Laboratory, Stockholm University, Solna, Sweden; 4 Department of Medical Biochemistry and Biophysics, Karolinska Institute, Stockholm, Sweden; 5 Present Address: Swiss Federal Institute of Technology Lausanne (EPFL), Lausanne, Switzerland

**Keywords:** Cell fate and cell lineage, Molecular neuroscience, Developmental neurogenesis

## Abstract

The mammalian brain contains many specialized cells that develop from a thin sheet of neuroepithelial progenitor cells. Single-cell transcriptomics revealed hundreds of molecularly diverse cell types in the nervous system, but the lineage relationships between mature cell types and progenitor cells are not well understood. Here we show in vivo barcoding of early progenitors to simultaneously profile cell phenotypes and clonal relations in the mouse brain using single-cell and spatial transcriptomics. By reconstructing thousands of clones, we discovered fate-restricted progenitor cells in the mouse hippocampal neuroepithelium and show that microglia are derived from few primitive myeloid precursors that massively expand to generate widely dispersed progeny. We combined spatial transcriptomics with clonal barcoding and disentangled migration patterns of clonally related cells in densely labeled tissue sections. Our approach enables high-throughput dense reconstruction of cell phenotypes and clonal relations at the single-cell and tissue level in individual animals and provides an integrated approach for understanding tissue architecture.

## Abstract (web summary)

Ratz et al. present an easy-to-use method to barcode progenitor cells, enabling profiling of cell phenotypes and clonal relations using single-cell and spatial transcriptomics, providing an integrated approach for understanding brain architecture.

## Main

High-throughput single-cell RNA sequencing (scRNA-seq) revealed hundreds of molecularly distinct cell types across the entire mouse and human nervous system^1–6^. However, a molecular understanding of the developmental origins of cell diversity remains limited, and a systematic analysis of lineage relationships is hampered by the low throughput of classical fate mapping techniques^7,8^. Advanced molecular tools have been used to record cell lineages^9–14^ and combined with scRNA-seq to generate fate maps in cultivated cells^15,16^, zebrafish^17–20^ and mice^14,16,21,22^. However, these technologies are not readily employed to uniquely label many progenitor cells in the mouse brain in vivo, and most approaches require tissue dissociation, although an in situ whole-transcriptome readout is crucial for studies of the nervous system where function arises from both differential gene expression and circuit-specific anatomy^23–26^.

Here we describe high-throughput clonal tracking and expression profiling of cells from the mouse forebrain using single-cell and spatial transcriptomics. We found two populations of fate-restricted progenitor cells present as early as embryonic day (E) 9.5 in the murine hippocampus. We discovered that microglia are generated from a limited number of progenitor cells that undergo massive clonal expansion as well as widespread migration across the mouse telencephalon. We recapitulated multiple migration patterns of progeny from brain progenitor cells using spatial transcriptomics of barcoded mouse brain tissue. Our findings demonstrate the utility of high-throughput clonal tracing in the mouse brain to provide molecular insights into brain development at the single-cell and tissue level.

## Results

### Unique labeling of progenitor cells with expressed barcodes

**Fig. 1:**
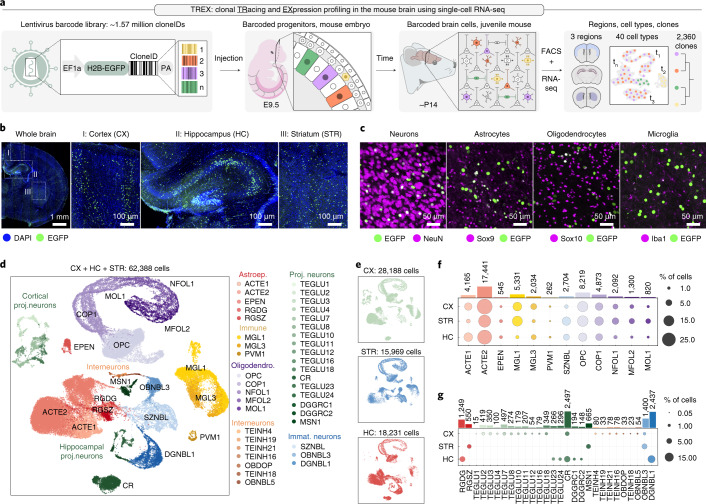
TREX enables simultaneous profiling of cell phenotype and clonality. **a**, Workflow for in vivo barcoding and profiling of brain cells. Microinjection of a highly diverse lentiviral barcode library into the ventricle of the developing mouse brain results in the expression of the barcode and EGFP in progenitor cells and their progeny. Each barcode serves as a unique cloneID that is inherited as progenitors differentiate into diverse brain cell types, and scRNA-seq is used to reveal the transcriptome and cloneID. We injected virus at E9.5 and isolated barcoded cells from three forebrain regions around P14 for scRNA-seq. **b**, Barcoding at E9.5 results in widespread and stable transgene expression in telencephalic regions, including cortex (CX), hippocampus (HC) and striatum (STR) of the postnatal brain (*n* = 3 brains). **c**, Barcoding at E9.5 results in stable transgene expression in all major cell types of the postnatal mouse brain (*n* = 3 brains). **d**, Visualization of identified cell classes using UMAP. In total, 62,388 single-cell transcriptomes were collected from three telencephalon regions and five brains (barcoded and non-injected controls) that were classified into 40 cell types. Capital black letters indicate a unique identifier for each cell type taken from www.mousebrain.org. Colors indicate six broader cell type classes: astroependymal (reds), immune (yellows), interneurons (oranges), projection neurons (greens), immature neurons (blues) and oligodendrocytes (purples). **e**, The same UMAP as in **d** split by regional origin of cells: CX (*n* = 28,188 cells, top), STR (*n* = 15,969 cells, middle) and HC (*n* = 18,231 cells, bottom). **f**,**g**, Dot plots showing fractions of cells in each class per region for cell types found in all three regions (**f**) and for cell types unique to one or two regions (**g**). Bar plots show total numbers of cells for each cell type. Source data

**Extended Data Fig. 1:**
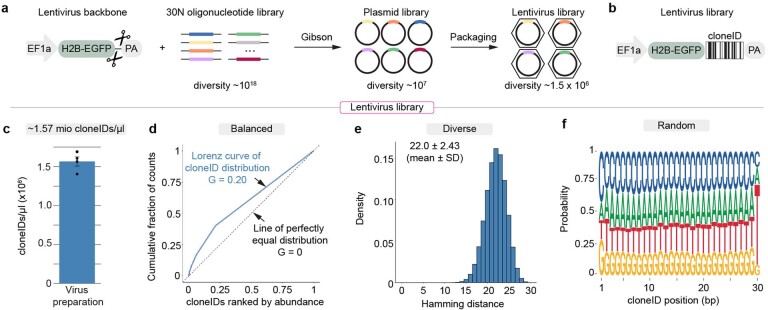
Generation and characterization of cloneID barcode libraries. a, A lentiviral backbone containing a strong human elongation factor 1 alpha (EF1a) promoter driving the expression of nuclear-localized enhanced green fluorescent protein via fusion to histone 2b (H2B-EGFP) was constructed based on LV-GFP. The EF1a-H2B-EGFP backbone was used for insertion of an amplified random 30N oligonucleotide library. The resulting plasmid library was subjected to lentiviral packaging to generate a lentivirus library with high functional titers greater than 10^9^ TU/ml. b, Each plasmid of the final library contains a unique random 30N barcode or cloneID downstream of the EGFP stop codon and known flanking sequences. c-f, Next generation sequencing was used for characterization of cloneID sequences in a typical lentivirus preparation (g-f). c, A total of 1,566,516 ± 120,080 cloneIDs/µl (mean ± SD, n = 4 virus preparations) were found in a typical lentivirus preparation. Graph represents average number of cloneIDs/µl, error bars represent SD, black dots represent individual data points corresponding to the number of cloneIDs per virus preparation. d, Lorenz curves (blue) were used to describe the inequality in the distribution of counts for all cloneIDs compared to the line of a perfectly equal distribution (dashed). The Gini coefficient (G), an overall measure of cloneID count inequality, is low for a typical lentivirus preparation indicating a uniform cloneID representation. e, The pairwise Hamming distance between all sequences for a random sample of cloneIDs (n = 10,000) was calculated for a representative lentivirus preparation demonstrating high cloneID sequence diversity. f, Sequence logo plot for a random sample of cloneIDs (n = 100,000) demonstrating that each cloneID is a random sequence based on similar probabilities for the occurrence of each of the four nucleotides (A, T, C, G) in the cloneID sequence at each of the 30 positions.

Here we present TREX, which enables TRacking and gene EXpression profiling of clonally related cells in the mouse brain by scRNA-seq (Fig. 1a). TREX relies on a diverse lentivirus library containing random 30-bp barcodes or cloneIDs downstream of a nuclear-localized enhanced green fluorescent protein (EGFP) driven by a strong, ubiquitous EF1a promoter (Extended Data Fig. 1a,b). A typical lentivirus preparation contained about 1.57 ± 0.12 × 10^6^ cloneIDs per microliter (mean ± s.d., *n* = 4) with a largely uniform representation (Gini index = 0.2) and high sequence diversity (Hamming distance = 22 ± 2.4, mean ± s.d., *n* = 10,000 random samples) (Extended Data Fig. 1c–f).

**Extended Data Fig. 2:**
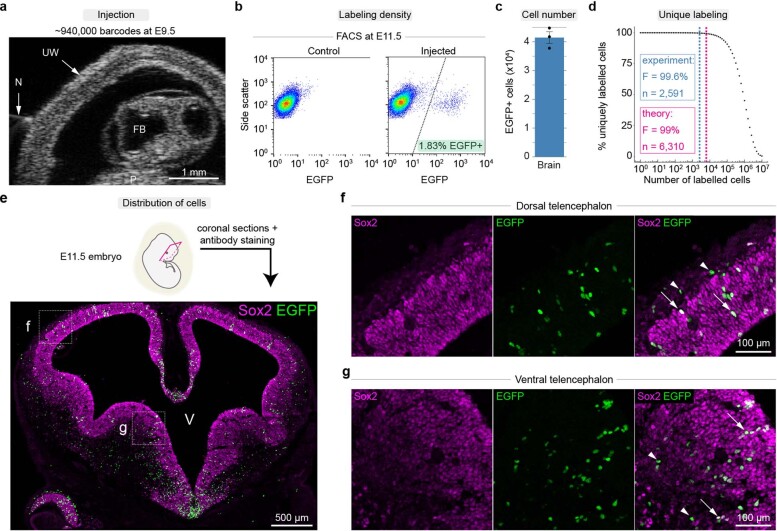
Unique labeling of mouse brain progenitors in vivo. a, Ultrasound image of mouse embryo at embryonic day (E) 9.5 showing injection needle (N), uterine wall (UW), placenta (P) and forebrain (FB). We injected a lentivirus volume of 0.6 µl corresponding to about 940,000 unique cloneIDs. b, Fluorescent activated cell sorting (FACS) of single cell suspensions prepared from a non-transduced control (left) and a representative lentivirus transduced (right) mouse brain two days after injection at E11.5. The fraction of EGFP+ cells upon lentivirus-mediated barcoding was 1.83 ± 0.25% (mean ± SD, n = 3 brains). c, Bar plot showing the total number of barcoded EGFP+ cells two days after injection. We counted a total of 2,279,750 ± 259,650 (mean ± SD, n = 3 brains) cells in single cell suspensions. Based on the fraction of labelled cells it can be estimated that a total of 41,450 ± 3,513 (mean ± SD, n = 3 brains) barcoded EGFP+ cells are present in a E11.5 mouse brain after virus injection at E9.5. Graph represents average number of cells per brain, error bars represent SD, black dots represent individual data points corresponding to the number of labelled cells per brain. d, Technically, the number of cloneIDs is sufficient to label 6,310 cells in a typical virus injection experiment (V = 0.6 µl, 0.94 × 10^6^ cloneIDs) with <1% barcode overlap between clones (magenta dashed line). Practically, around 2,600 cells are labelled in a typical experiment (blue dashed line) hence 99.6% of these cells were uniquely labelled with a cloneID. e, Top, Schematic of E11.5 mouse embryo used for cryosectioning following lentivirus injection into the E9.5 brain. Bottom, Representative image of a coronal E11.5 brain section showing Sox2+ neuronal progenitor cells located primarily in the ventricular zone as well as lentivirus transduced EGFP+ cells distributed throughout the neural tube tissue. V, ventricle. f, g, Sox2+/EGFP+ double positive progenitor cells (arrows) and their Sox2-/EGFP+ daughter cells (arrow heads) located in the dorsal (f) and ventral (g) telencephalon, respectively. e-g, n = 3 brains.

To label individual progenitor cells in vivo, we used in utero microinjection of lentivirus into the ventricular system of the mouse forebrain at E9.5 (Extended Data Fig. 2a). We injected about 0.6 µl of EGFP-cloneID virus corresponding to 0.94 × 10^6^ unique cloneIDs and which resulted in labeling of 1.8 ± 0.25% of all cells (mean ± s.d., *n* = 3) or a total of 41,000 ± 3,500 cells (mean ± s.d., *n* = 3) per E11.5 mouse brain (Extended Data Fig. 2b,c). We estimated that the initial number of labeled progenitors was around 2,600 cells at E9.5 and that 99.6% of cells were uniquely labeled with a cloneID (Extended Data Fig. 2d).

Barcoded EGFP^+^ cells were mostly evenly distributed throughout the E11.5 neuroepithelium and included Sox2^+^ radial glia progenitors lining the ventricular zone as well as their Sox2^−^ daughter cells (Extended Data Fig. 2e–g). Long-term EGFP-cloneID expression was maintained in all major cell types of the juvenile mouse brain, and labeled cells were found in various regions (Fig. 1b,c). In conclusion, we present a highly diverse lentivirus library suitable for heritable and brain-wide labeling of thousands of mouse brain progenitors with unique barcodes.

### Molecular identity of barcoded brain cells

To determine the molecular identity of labeled cells, we dissected brains from 2-week-old mice and isolated all EGFP^+^ barcoded cells separately from cortex (CX), striatum (STR) and hippocampus (HC) for scRNA-seq (Supplementary Fig. 1a–d). We collected transcriptome profiles of 65,160 cells from these three regions per brain from four barcoded and one non-injected control brain. Graph-based clustering revealed five main clusters corresponding to astroependymal cells, immune cells, neurons and oligodendrocyte and vascular cells (Supplementary Fig. 1e). Both control and barcoded samples showed a similar cell type composition except for vascular cells, which represented 16.6% of cells in the control dataset and less than 0.5% of barcoded cells (Supplementary Fig. 1f). Blood vessels only begin to sprout into the ventrolateral brain at E9.5 (ref. ^27^), which results in a low number of cells that can be labeled at the time point of injection. We, therefore, removed the cluster of vascular cells from all datasets and kept a final of 62,388 single-cell profiles with a mean of 5,444 transcripts and 2,255 genes detected per cell (Supplementary Fig. 1g–l).

We performed subclustering for each major cell type from all brain regions and assigned each cell subclass a unique mnemonic identifier based on an existing mouse brain atlas^2^ (Supplementary Fig. 2). We found 40 molecularly defined cell classes, including projection neurons (*n* = 17), GABAergic interneurons (*n* = 7), immature neuronal cells (*n* = 3), astroependymal cells (*n* = 5), oligodendrocyte lineage cells (*n* = 5) and immune cells (*n* = 3) (Fig. 1d). We collected the highest number of cells from CX (*n* = 28,188 cells), followed by HC (*n* = 18,231 cells) and STR (*n* = 15,969 cells) (Fig. 1e–g). We compared gene expression profiles and total cell type composition between barcoded and non-injected samples, which indicated that lentivirus-mediated barcoding does not perturb cell physiology (Supplementary Fig. 3). Together, these data show the utility of TREX for barcoding progenitor cells in the developing brain and profiling the identity of their progeny at a postnatal stage.

### Barcode expression metrics across cell types

**Fig. 2:**
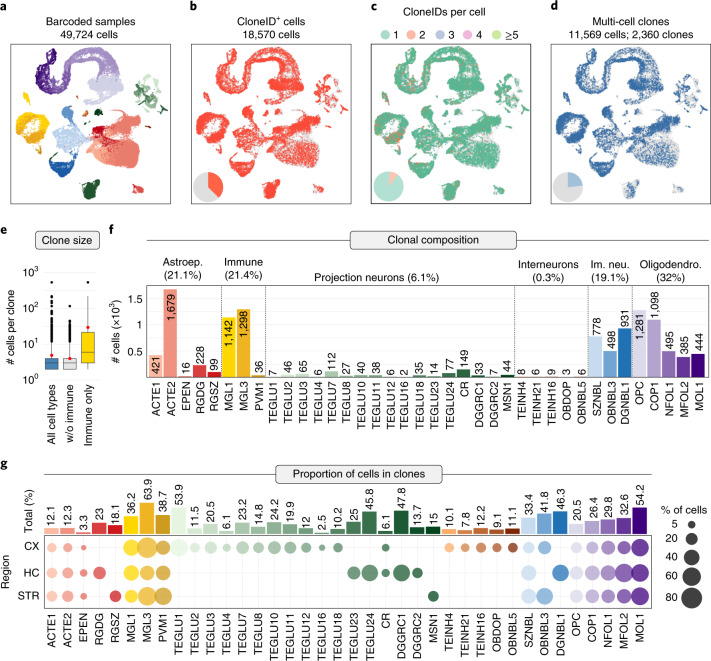
Clone reconstruction across cell types and regions in the mouse telencephalon. **a**–**d**, UMAP visualizations of all cells isolated from barcoded samples grouped by cell type (**a**), cloneID-expressing cells (**b**), number of cloneIDs per cell (**c**) and cells in multi-cell clones (**d**). **e**, Box plots showing average clone sizes (number of cells per clone). The average clone size varies depending on cell type and is 4.9 ± 0.3 cells per clone (mean ± s.e.m., *n* = 2,360 clones) when considering all cell types (left), 4 ± 0.1 cells per clone (mean ± s.e.m., *n* = 2,276 clones) when considering only neuroectoderm-derived cells (middle) and 29.5 ± 7.6 cells per clone (mean ± s.e.m., *n* = 84 clones) when considering only mesoderm-derived immune cells (right). For each plot, the center line represents the median; the bounds of the box represent the interquartile range; and the whiskers represent the lower and upper limits. Black dots represent outliers, and red dots represent the average clone size. **f**, Bar plots showing total number of cells in clones for each cell type. **g**, Dot plots and bar plots displaying the total proportion of cells in clones for each cell type (top) and per region (bottom). Source data

**Extended Data Fig. 3:**
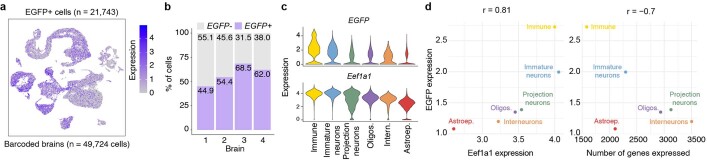
EGFP expression metrics from synthetic EF1a promoter. **a**, UMAP embeddings and normalized *EGFP* expression levels in all cells (n = 49,724) isolated from postnatal mouse brains that were injected with EF1a-H2B-EGFP-cloneID libraries at E9.5. A total of 21,743 cells contained at least one *EGFP* transcript. **b**, Bar plots showing the proportion of *EGFP* transcript positive cells per brain ranging from 44.9% to 68.5% (57.4% ± 10.2%, mean ± SD, n = 4 brains). **c**, Violin plots showing *EGFP* (top) and *Eef1a1* (bottom) expression levels for each broad cell class sorted by decreasing average *EGFP* expression. **d**, Left, scatter plot showing a high Pearson correlation (r = 0.81) between average expression levels for *EGFP* and *Eef1a1* for each cell class indicating that the synthetic EF1a promoter recapitulates endogenous *Eef1a1* expression patterns albeit at lower levels. Right, scatter plot showing a low Pearson correlation (r = −0.7) between *EGFP* expression levels and average number of genes expressed for each cell class.

To specifically study barcoded cells, we removed cells from non-injected control samples from the full dataset and focused on the 49,724 cells isolated from four barcoded mouse brains (Fig. 2a). We detected EGFP transcripts in a total of 21,743 cells (43.7% of all cells), with highest average expression in immune cells and lowest average expression in astroependymal cells (Extended Data Fig. 3a–c). The number of EGFP transcripts per cell class was correlated (Pearson’s *r* = 0.81), with elongation factor 1-alpha 1 (Eef1a1) levels indicating that transgene expression under the synthetic EF1a promoter recapitulates endogenous Eef1a1 expression patterns, albeit at lower levels (Extended Data Fig. 3d).

**Extended Data Fig. 4:**
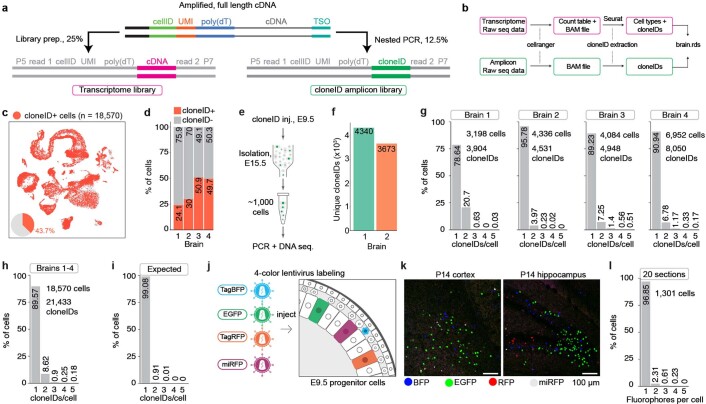
cloneID sequencing and expression metrics. **a**, cloneID sequences are obtained from a transcriptome library and an amplicon library. To enrich for cloneID sequences, we prepared a second library using a targeted, nested PCR approach from full length cDNA obtained during the first steps of transcriptome library preparation. **b**, Simplified workflow for cloneID extraction from amplicon and transcriptome raw sequencing data. We used cellranger (10X Genomics) for raw sequence alignment of both transcriptome and amplicon data and Seurat for analysis of transcriptome data (cell filtering, clustering, etc). We then extracted genetic barcodes (cloneIDs) from aligned sequences (BAM file) corresponding to transcriptome and amplicon data for high quality cells. **c**, UMAP embeddings for all cells (n = 49,724) isolated from barcoded brains and cloneID containining cells highlighted in red (n = 18,570). **d**, Bar plots showing the proportion of cloneID positive cells per brain ranging from 24.1% to 50.9% (38.4% ± 11.8%, mean ± SD, n = 4 brains). **e**, **f**, Targeted DNA sequencing of cloneIDs does not reveal more cloneIDs per cell than expected from scRNA-seq. Bulk DNA sequencing of cloneID sequences from EGFP+ cells isolated six days after lentivirus library injection into the E9.5 mouse brain (c) revealed that the number of cloneIDs ranges from 3,673 to 4,340 unique sequences for 4,000 cells (d). **g, h**, Most cells express only one cloneID upon lentiviral barcoding. Histogram showing the number of cloneIDs per cell for all cloneID positive cells from each brain (g) separately and a summary of all brains (h). **i**, Expected distribution of cloneID copy numbers among transduced cells assuming an idealized model of transduction. Based on the previously observed transduction rate of 1.83%, we expected that >99% of transduced cells contain only cloneID. **j-l**, Most cells express only one fluorophore upon 4-color lentivirus injection. Multicolor labeling of E9.5 progenitor cells with a high titer lentivirus library encoding four different fluorophores (**j**) rarely results in co-expression of more than one fluorophore in the same cell of the postnatal mouse brain (**k, l**) confirming that cloneIDs are quantitatively captured using single cell transcriptomics.

We extracted cloneIDs directly from single-cell transcriptome data as well as targeted amplicon libraries and found a total of 21,433 cloneIDs in 18,570 cells (37.3% of all cells) (Fig. 2b and Extended Data Fig. 4a–d). We captured cloneIDs for most cell types except for one very rare type of interneuron, TEINH18. The average number of cloneIDs per cell was similar when using scRNA-seq or bulk DNA sequencing of barcoded cells (Extended Data Fig. 4e,f), suggesting that cloneID capture is quantitative using single-cell transcriptomics.

Although most cloneID^+^ cells (89.6%) across all brains expressed only one cloneID (Fig. 2c), the proportion of such cells varied among brains and ranged from 78.6% to 95.8%, with the remaining fraction of cells expressing multiple cloneIDs (Extended Data Fig. 4g,h). Based on the transduction rate of 1.8% and an idealized transduction model^28^, we expected that 99.08% of cells contain one cloneID and 0.92% of cells contain two or more cloneIDs (Extended Data Fig. 4i). The observed deviation from the theoretical cloneID copy number distribution per brain is not due to undetected doublets in scRNA-seq (Extended Data Fig. 7j–l) but can be attributed to increased transduction rates of local progenitor cells due to position and/or differential expression of receptors required for lentivirus entry (Supplementary Figs. 4 and 5). In summary, all major brain cell types were represented among barcoded cells, and most cells express a single cloneID.

### Clonal relationships across forebrain regions

**Extended Data Fig. 5:**
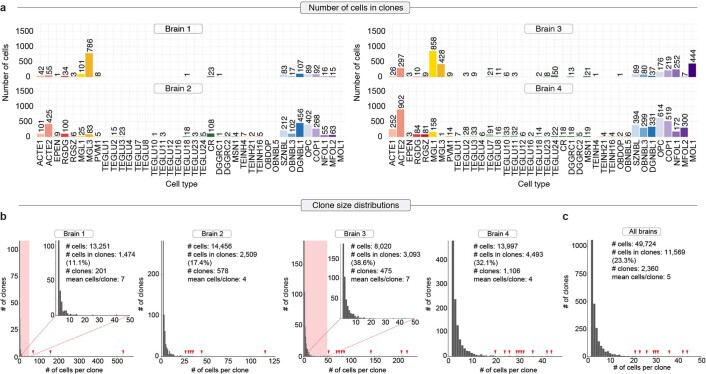
Clone calling metrics for each brain. **a**, Bar plots showing the number of cells contained in clones for cell type and brain. **b**, Histograms and key summary metrics showing the clone size distribution for each brain separately. Shown is the entire distribution of clone sizes for clones containing at least two cells and a magnification (red area) for clone sizes between 2 and 50 cells per clone for brains 1 and 3 that contained a wide range of clone sizes. Red arrow heads indicate the size of rare clones occurring at low frequency. **c**, Histogram and key summary metrics showing the clone size distribution for all clones reconstructed from all brains. Because most clones contained less than 50 less, we displayed only clones ranging from 2 to 50 cells in the histograms.

We identified clonally related cells based on the Jaccard similarity of cloneIDs for each pair of cloneID-containing cells^15^, and we defined clones as groups of two or more related cells. We reconstructed 2,360 clones containing 11,569 cells (23.3% of all cells; Fig. 2d) with an average size of 4.9 ± 0.3 cells per clone (mean ± s.e.m.). The number of clones per brain ranged from 201 to 1,106 (11.1% to 38.6% of all cells per brain; Extended Data Fig. 5). Interestingly, clones containing mesoderm-derived myeloid cells were about 7.4 times larger than those with neuroectoderm-derived cells and contained 29.5 ± 7.6 cells per clone (mean ± s.e.m., *n* = 84 clones) compared to 4 ± 0.1 cells per clone (mean ± s.e.m., *n* = 2,276 clones), respectively (Fig. 2e). This difference in clone size probably reflects the massive proliferation of brain macrophages required to colonize the entire central nervous system (CNS) after only a small number of precursors enter the brain before closure of the blood–brain barrier around E13 (ref. ^29^).

**Extended Data Fig. 6:**
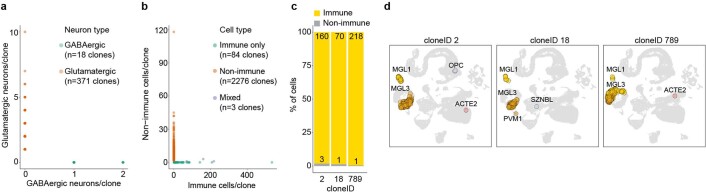
Cell types of different origin do not share cloneIDs. **a**, Scatter plot showing the number of cells in clones containing inhibitory neurons (x-axis, green, n = 18 clones) or excitatory neurons (y-axis, red, n = 371 clones). We never observed a shared cloneID between these two cell types. **b**, Scatter plot showing the number of cells in clones containing mesoderm-derived immune cells (x-axis, green, n = 84 clones) or neuroectoderm-derived non-immune cells (y-axis, red, n = 2,276 clones). We rarely observed mixed clones (n = 3 clones) containing cells of both lineages. **c**, Bar plot showing proportion and total number of cells per type for the 3 mixed clones from (b). Only very large immune clones contain very few non-immune cells indicating a very low rate of contamination. **d**, UMAP visualizations for the 3 mixed clones from (b). The five neuroectoerm-derived cell types observed together with 448 immune cells were two protoplasmic astrocytes (ACTE2), two oligodendrocyte precursor cells (OPC) and one intermediate neuronal progenitor cells (SZNBL).

To estimate the potential error associated with clone reconstruction, we quantified how often cell types that arise from different progenitors shared the same cloneID. We found that clones containing cortical excitatory neurons (*n* = 371 clones) or inhibitory neurons (*n* = 18 clones), which are known to have separate developmental origins^30^, never shared the same cloneID (Extended Data Fig. 6a). Second, among 84 clones containing 2,481 mesoderm-derived microglia or perivascular macrophages, only three clones with a total of 453 cells shared a cloneID with five neuroectoderm-derived cells (Extended Data Fig. 6b–d), and we removed these cells from the respective clones. These data suggest a low error rate of about 0.2% (five of 2,481 cells) that could be related to clone size and cell type, because only large immune clones contained neuroectoderm-derived cells, or to non-unique cloneID labeling. We found that cloneID removal from cell types that express multiple cloneIDs results in ‘lumping’ errors (Supplementary Fig. 6). This is expected because the co-expression of two or more distinct cloneIDs per cell leads to a higher combinatorial diversity^15^, thus reducing the error associated with clone reconstruction. Finally, there was a high correlation (Pearson’s *r* = 0.99) between cloneID frequency and the number of cells with a cloneID in distinct clones, indicating that there is no preferential uptake of certain barcodes among progenitor cells (Supplementary Fig. 7).

Cell types most often represented in clones were oligodendrocyte subtypes (3,703 cells, 32%), followed by immune cells (2,476 cells, 21.4%), astroependymal cells (2,443 cells, 21.1%), immature neuronal cells (2,207 cells, 19.1%), projection neuron types (708 cells, 6.1%) and interneurons (32 cells, 0.3%) (Fig. 2f). Except for one type of interneuron, TEINH19, we captured clonal information for all cell types that also contained a cloneID. Cell types containing the highest proportion of cells in clones were cortical and striatal microglia (MGL3), of which 77.5% and 60.6%, respectively, of all sampled MGL3 cells per region were represented in clones (Fig. 2g). The lowest proportions of cells represented in clones were observed for ependymal (EPEN) cells (1.3–4.9% of EPEN cells across all regions), TEGLU16 piriform pyramidal neurons (2.5%) and TEINH21 inhibitory neurons (7.8%) in the CX. In line with our previous observation, we found a high correlation (Pearson’s *r* = 0.57) between the number of cells in clones and barcode expression level for each cell type (Supplementary Fig. 8). In conclusion, we captured clonal information about most cell types in different regions of the mouse telencephalon and demonstrated that reconstruction of clonal relationships using TREX has a very low error rate.

### Regional distributions of clonally related cells

**Fig. 3:**
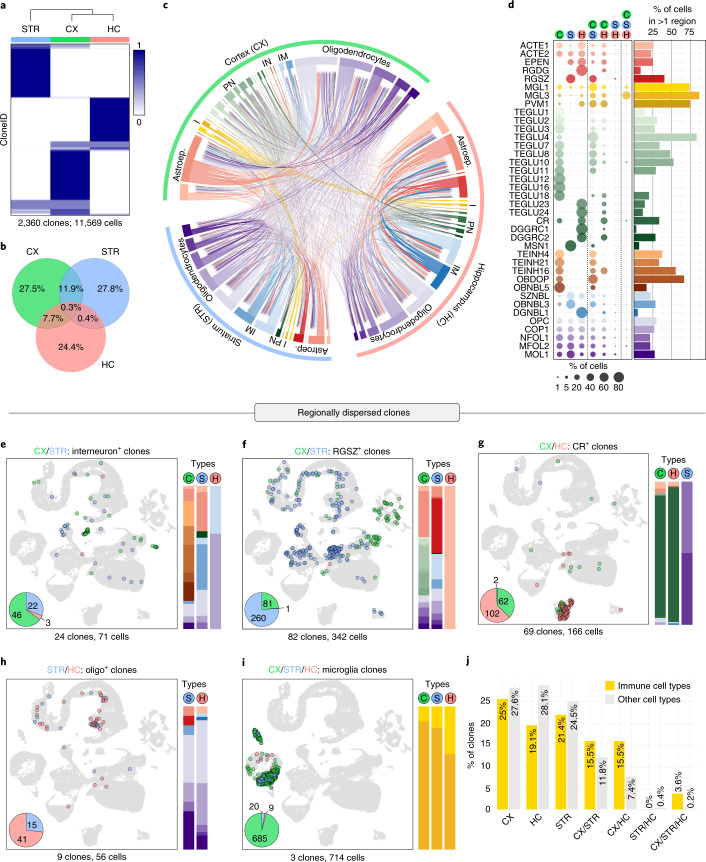
Regional distribution of clonally related cells across the telencephalon. **a**,**b**, Most cells belonging to the same clone were restricted to a single brain region. For each cloneID (rows), the proportions of cells within each region (columns) were calculated, scaled by row and colored as shown in the figure (**a**). The fraction of cells per clone belonging to one region (CX, STR or HC), two regions (CX/STR, CX/HC or STR/HC) or all three forebrain regions (CX/STR/HC) was determined and displayed as a Venn diagram (**b**). All 2,360 clones and a total of 11,569 cells were considered. CX, cortex (green); STR, striatum (blue); HC, hippocampus (red). **c**, *Circos* plot displaying shared cloneIDs among all cell classes (inner segments) across CX, STR and HC (outer segments). For each cell pair, the number of shared cloneIDs is indicated by the width of the link, and the color of each link represents cell type. **d**, Cell types associated with dispersed clones were identified by determining the cell type composition of clones spread across multiple telencephalon regions relative to the total number of cells in clones for each cell type. The bar plot summarizes the proportion of cells in clones spread across multiple regions. **e**–**i**, Examples of cell types in clones dispersed across multiple regions. Each example contains a UMAP visualization of cells, a pie chart displaying the number of cells per region and bar plots to illustrate the cell type composition of clones. Selected clones dispersed across the anatomical boundaries between CX and STR (**e**,**f**), CX and HC (**g**), STR and HC (**h**) and all three regions (**i**) are displayed. **j**, Clonally related immune cells (MGL1, MGL3 and PVM1) disperse more frequently across telencephalon regions compared to neuroectoderm-derived cells. The fraction of clones containing clonally related cells found in one region (CTX, STR, HC), two regions (CX/STR, CX/HC, STR/HC) or three regions (CX/STR/HC) is shown. Source data

Because we isolated barcoded cells from CX, STR and HC, we asked how often clonally related cells spread across these areas. By calculating the proportions of cells across each forebrain region for each cloneID, we observed that the cells of 1,880 clones (79.7%) accumulated in a single region (Fig. 3a,b). Clonal dispersion of progenitors across more than one region was less frequent and was observed for 282 clones (11.9%) spreading across CX and STR as well as CX and HC (182 clones, 7.7%) but rarely between STR and HC (nine clones, 0.4%) or all three regions (seven clones, 0.3%). This indicates that most clonally related cells show limited regional dispersion across the mouse telencephalon.

To assess which cell types were associated with dispersed clones, we determined the cell type composition of clones spread across multiple forebrain regions relative to the total number of cells in clones for each cell type (Fig. 3c,d and Supplementary Fig. 9). We found that clonally related cells that crossed the CX/STR boundary often contained inhibitory neurons such as medial ganglionic eminence (MGE)-derived neurogliaform cells (TEINH16) in the CX (Fig. 3e). Inhibitory neurons shared a cloneID with medium spiny neurons (MSN1) and gray matter astrocytes (ACTE2) in the STR as well as neuronal intermediate progenitor cells (SZNBL) and oligodendrocyte subtypes in both STR and HC. These data suggest that MGE-derived cortical interneurons are generated by distinct progenitor cells and revealed that individual progenitors can give rise to both neurons and oligodendrocytes. Also, subventricular zone neural stem cells (RGSZ) in the STR often shared a cloneID with cells such as gray matter astrocytes (ACTE2), layer 2/3 excitatory neurons (TEGLU7) and all oligodendrocyte subtypes in the CX (Fig. 3f). This example of transcriptional divergence demonstrates a direct clonal relationship between E9.5 progenitor cells that generate RGSZ neural stem cells and those that produce neurons and glia cells for the other regions of the telencephalon during embryonic development.

**Extended Data Fig. 7:**
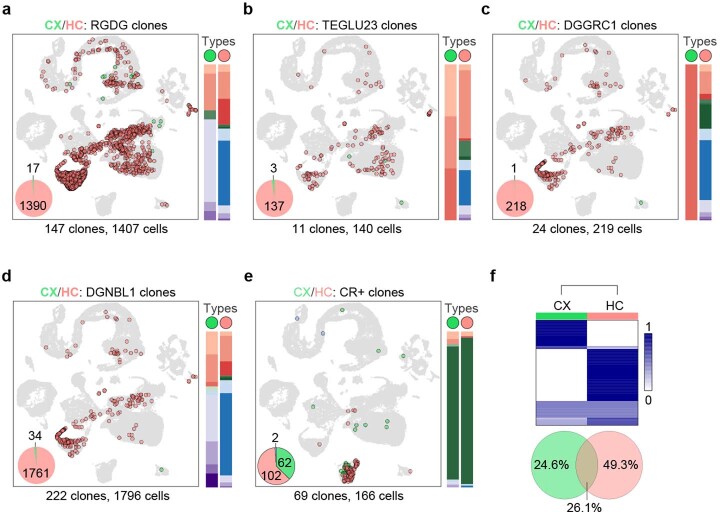
Transcriptional convergence of Cajal-Retzius cells. **a-d**, Most cell types that were specifically found in the hippocampus shared a cloneID with multiple other cell types in hippocampus, but rarely with other cell types in cortex indicating an early segregation of progenitor fields for both regions. Examples for hippocampal cell types are shown for adult neural stem cells in the subgranular zone (a, RGDG), excitatory CA3 neurons (b, TEGLU23), dentate gyrus granule neurons (c, DGGRC1) and granule neuroblasts (d, DGNBL1). **e**, An exception to this pattern are clones with Cajal-Retzius cells (CR). Such clones rarely contained other cell types and often shared a cloneID with CR cells in cortex indicating that the progenitors from disparate embryonic fields converge in their differentiation to produce transcriptionally similar cells. **f**, Heatmap showing the proportions of CR cells across both regions for each cloneID. We observed that the 24.6% of cloneIDs accumulated in cortex (CX) only, 49.3% in hippocampus (HC) only and 26.1% are spread across both CX and HC.

Many cell types that were specifically found in the HC shared a cloneID with multiple other cell types in HC but rarely with other types in CX, indicating an early segregation of progenitor fields for both regions (Extended Data Fig. 7a–d). However, clones with Cajal–Retzius (CR) cells were an exception that rarely contained other cell types and often shared a cloneID with CR cells in CX (Fig. 3g). We quantified the proportions of CR cells across both regions for each cloneID and observed that 24.6% of cloneIDs accumulated in CX, 49.3% in HC and 26.1% spread across both CX and HC (Extended Data Fig. 7e,f). CR cells are among the first-born neurons critical for brain development, our data confirm that these cells originate from three distinct sites in the brain^31^ and further indicate that the progenitors from disparate embryonic fields converge in their differentiation to produce transcriptionally similar cells.

The anatomical boundary between HC and STR was rarely crossed (Fig. 3h), and cell types associated with such clones were mostly oligodendrocyte types such as oligodendrocyte precursor cells (OPCs) and committed oligodendrocyte precursors (COP1). This suggests that oligodendrocytes in both HC and STR are derived from a common progenitor most likely located in the ventral forebrain, which generates OPCs that subsequently migrate widely into all parts of the telencephalon before differentiating^32^.

Finally, clonally related immune cells comprising microglia (MGL1 and MGL3) and perivascular macrophages (PVM1) showed a widespread regional dispersion and crossed anatomical boundaries among CX, STR and HC 1.3-fold to nine-fold more often than neuroectoderm-derived clones (Fig. 3i,j). This suggests that myeloid progenitors and their progeny undergo extensive migration to populate large areas of the forebrain.

### Fate distributions of clonally related cells

**Fig. 4:**
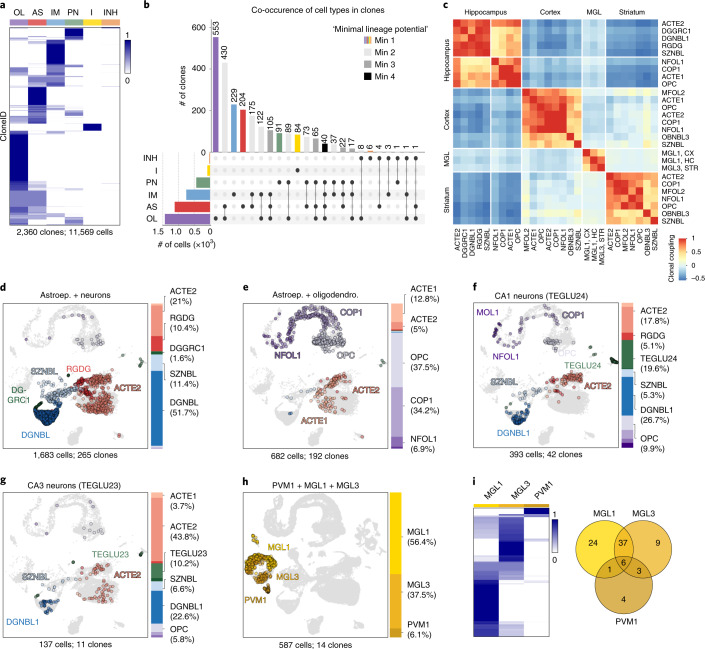
TREX reveals fate-biased progenitors of neuroectodermal and myeloid origin. **a**, Heat map showing the proportions of each major cell type (columns) per cloneID (rows). For each cloneID, the proportions of cells within each cell type were calculated, scaled by row and colored as shown in the figure. OL, oligodendrocyte; AS, astroependymal cell; IM, immature neuron; PN, projection neuron; I, immune cell; INH, inhibitory interneuron. **b**, *UpSet* plot showing co-occurrence of cell types in the same clone. Bar plot shows the number of clones containing a particular combination of cell types, and each bar is a different combination. The graphical table underneath the bar plot displays what those combinations are. Because not all cells from a clone have been isolated, the progenitor cell of origin is ‘minimally uni-potent’ etc. **c**, Heat map showing correlation between clonal coupling scores defined as the number of shared cloneIDs relative to randomized data for each pair of cell types. High correlation values indicate a clonal relationship between cell types and a common progenitor cell. Clustered using the complete linkage method. **d**,**e**, Precursor cells in the hippocampus neuroepithelium are biased to generate one of two fates. UMAP visualizations and bar plots for clones containing astroependymal cells (ACTE2 and RGDG) and neuronal cells (DGNBL, SZNBL and DGGRC1) associated with fate 1 (**d**) as well as clones containing astroependymal cells (ACTE1 and ACTE2) and oligodendrocyte subtypes (OPC, COP1 and NFOL1) associated with fate 2 (**e**). **f**,**g**, Early fate specification of CA1 and CA3 excitatory neurons. Clones containing CA1 (TEGLU24) neurons never contained CA3 (TEGLU23) neurons (**f**) and vice versa (**g**). Otherwise, these clones contained the same cell types. **h**,**i**, Macrophages of the CNS parenchyma and CNS borders share the same precursor cell. UMAP visualization and bar plot for all clones that contained perivascular macrophages (PVM1, **h**). Heat map showing the proportion of cells classified as MGL1, MGL3 or PVM1 for each cloneID and a Venn diagram displaying the number of clones containing cells of one, two or all three types (**i**) considering all 84 clones with 2,476 immune cells. Source data

We investigated the distribution of cloneIDs across cell types by calculating the proportions of cells within each major cell class for each cloneID. We found that immune cells (*n* = 84 clones) consisting of microglia and perivascular macrophages constitute a separate lineage as expected (Fig. 4a). Of the remaining 2,276 neuroectoderm-derived clones, a total of 1,193 clones (52.4%) contained at least two different cell types (Fig. 4b). The remaining 1,083 neuroectoderm-derived clones contained only one of the five major cell types, and such clones were also observed among the largest clones (Supplementary Fig. 10). Although this might suggest that many lineage-restricted progenitor cells exist in the E9.5 mouse neuroepithelium, we cannot conclude that a strictly ‘uni-potential’ progenitor was present during barcoding, because only a small sample of its progeny had been isolated.

To systematically assess lineage relationships among subclasses of all cell types, we investigated the probability of recovering shared cloneIDs from all pairs of profiled cells in the mouse brain. We calculated the clonal coupling score, defined as the number of shared cloneIDs relative to randomized data^20^, yielding values that range from positive (related cells) to negative (unrelated cells) for each brain (Supplementary Fig. 11). To summarize the data for all brains, we focused on the 27 cell types found in clones with at least three cells per clone across all four brains and determined the pairwise correlation between coupling scores. Hierarchical clustering of the pairwise correlations revealed four distinct groups of clonally related cells corresponding to diverse cell types of the cortex, hippocampus and striatum as well as microglia from all three regions (Fig. 4c). These results corroborated our previous observations regarding the limited clonal dispersion of most neuroectoderm-derived cell types across the mouse telencephalon.

We observed a strong clonal coupling in the HC between neuronal and astroependymal cells (fate 1) as well as between astroependymal cells and oligodendrocytes (fate 2), indicating that these cells originate from two fate-biased pools of progenitor cells. We found that 265 clones containing 1,683 cells were biased toward fate 1 (Fig. 4d) and consisted mainly of neuronal cell types such as dentate gyrus neuroblasts (DGNBL1, 51.7%), neuronal intermediate progenitor cells (SZNBL, 11.4%) and granule neurons (DGGRC1, 1.6%) as well as astroependymal cells, including gray matter astrocytes (ACTE2, 21%) and radial glia-like cells (RGDG, 10.4%). A total of 192 clones with 682 cells were biased toward fate 2 (Fig. 4e) and contained mainly oligodendrocyte subtypes such as OPCs (37.5%), committed oligodendrocyte precursors (COP1, 34.2%) as well as astroependymal cells, including white matter astrocytes (ACTE1, 12.8%) and gray matter astrocytes (ACTE2, 5%). One population of progenitor cells, fate 1, likely corresponds to the embryonic precursors of adult neural stem cells^33^ that are biased to generate astroependymal cells and dentate granule neurons as early as E9.5. The second precursor cell population, fate 2, mainly contains oligodendrocyte subtypes and could represent a major source of hippocampal glia cells involved in myelin formation and maintenance.

We also investigated the cloneID distribution across cell types that were not included in the clonal coupling analysis, because they were not isolated from all four brains and/or they were not contained in clones with at least three cells per clone. Interestingly, we never observed hippocampal CA1 (TEGLU24) and CA3 (TEGLU23) excitatory neurons in the same clone that otherwise contained identical cell types (Fig. 4f,g). Because the number of clones containing at least one CA1 or CA3 neuron was small (42 clones with 77 CA1 cells and 11 clones with 14 CA3 cells), we cannot exclude that these cells share a common progenitor. However, our observations are in agreement with previous studies about the early specification of CA field identity^34^ and might indicate a fate specification (or at least fate bias) as early as E9.5.

We investigated the clonal relationships between microglia in the brain parenchyma (MGL1 and MGL3) and perivascular macrophages (PVM1) located at CNS borders. We found that ten of 14 clones (*n* = 587 cells) that contained PVM1 cells also contained one or both microglia subtypes (Fig. 4h,i). Compared to 331 MGL1 cells (56.4%) and 220 MGL3 cells (37.5%), these clones contained only 36 PVM1 cells (6.1%). Because barcode expression levels and proportion of cells in clones were similar for MGL1, MGL3 and PVM1 (Fig. 2g and Supplementary Fig. 8), this observation indicates that the common progenitor for all three cell types largely generates microglia and few perivascular macrophages. Although it has been established that microglia are derived from mesodermal progenitors^35,36^, it has been shown only recently that the same early embryonic precursors also generate perivascular macrophages^37,38^. Our results are in line with this observation and further revealed that microglia are generated in much larger numbers than perivascular macrophages from a common progenitor cell.

### Spatial profiling of transcriptomes, cell types and clones

**Fig. 5:**
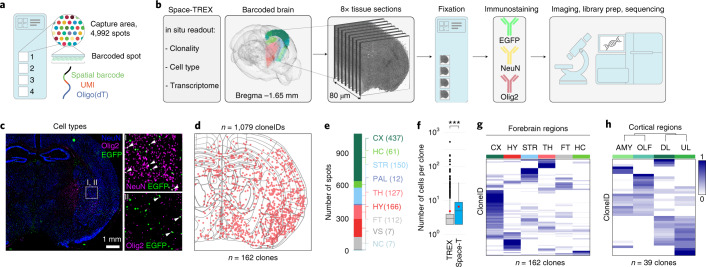
Space-TREX enables simultaneous profiling of transcriptomes and clones in situ. **a**, Glass slide layout used for ST. Each capture area contains 4,992 barcoded spots with a diameter of 55 µm and a center-to-center distance of 100 µm. Each spot contains spatially barcoded oligonucleotides that bind mRNA released from the tissue, enabling gene expression profiling in situ. **b**, Overview of Space-TREX. Adjacent 10-µm sections (*n* = 8) were collected, fixed and incubated with fluorophore-conjugated antibodies recognizing EGFP (barcoded cells), NeuN (neurons) and Olig2 (oligodendrocytes), followed by imaging, library preparation and sequencing. **c**, Image of one stained section (left, *n* = 4 sections) and a zoom-in showing barcoded neurons (I, white arrowheads) and barcoded oligodendrocytes (II, white arrowheads). **d**, 1,321 cloneIDs were extracted from all sections, and 1,079 cloneIDs were contained in 162 clones. These cloneIDs are displayed as red dots projected onto a digital brain section containing anatomical reference outlines. **e**, Number of cloneID^+^ spots per brain region. **f**, Clones reconstructed using Space-TREX (*n* = 162 clones) contain a significantly higher average number of cells compared to TREX (*n* = 2,360 clones) (Welch two-sample *t*-test, *t* = 3.3205, *df* = 344.2, *P* = 0.0009948; 95% confidence interval is 0.7–2.8). For each plot, the center line represents the median; the bounds of the box represent the interquartile range; and the whiskers represent the lower and upper limits. Black dots represent outliers, and red dots represent the average clone size. **g**, Regional distribution of clonally related cells across the forebrain region reveals patterns of clonal dispersion. Regions containing more than 20 cloneIDs were considered. For each cloneID, the proportions of cells within each region were calculated, scaled by row and colored as shown in the legend. CTX, cortex; HY, hypothalamus; STR, striatum; TH, thalamus; FT, fiber tracts; HC, hippocampus. **h**, High-resolution spatial mapping of clonal dispersion across cortical regions reveals progenitors with a bias to generate cells of either amygdalar (AMY) and olfactory (OLF) areas or upper layer (UL) and deep layer (DL) cells. Source data

**Extended Data Fig. 8:**
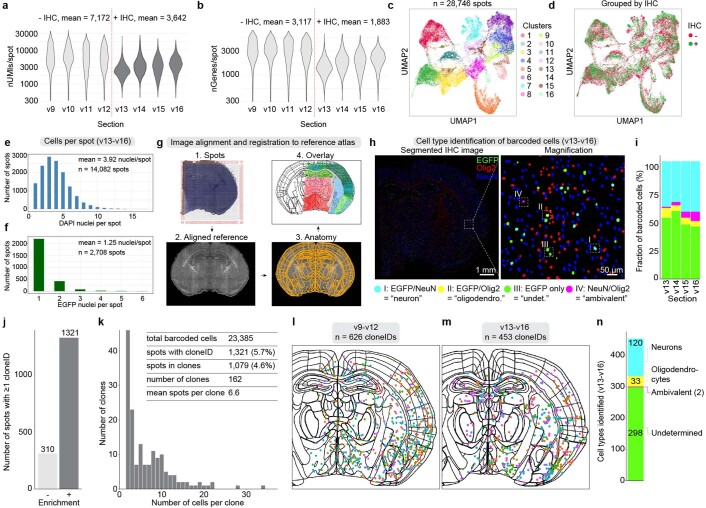
Space-TREX enables simultaneous profiling of gene expression, clonal tracking and phenotyping of cell types in situ. A total of eight 10 µm thick brain sections ‘v9-v16’ were used. Four sections ‘v9-v12’ were processed using the regular spatial transcriptomics workflow and four sections ‘v13-v16’ were used for spatial transcriptomics coupled to immunohistochemical (IHC) staining. **a, b**, The average number of unique molecular identifiers (UMIs) per spot (a) and the average number of genes detected per spots (b) is lower when a section undergoes IHC staining. This is most likely caused by re-folding and re-activation of RNA degrading enzymes in aqueous buffers used for IHC following methanol fixation. **c**, A total of 28,746 spots were sequenced from all eight sections that could be grouped into 16 distinct clusters. **d**, Spots belonging to sections that underwent IHC clustered together with spots from regularly processed sections indicating that IHC staining contains similar molecular information. **e, f**, Histograms showing the number of DAPI+ cells per spot (e) and EGFP+ cells per spot (f) as quantified from immunostained sections v13-v16. **g**, Workflow illustrating image processing for alignment to standardized anatomical reference atlas using WholeBrain. The resulting output contains both the coordinates of spatial transcriptomics spots within the Allen mouse brain reference atlas and each spot is displayed using the regional color code from this reference. **h**, Cell types in EGFP/NeuN/Olig2 triple immunostained tissue sections v13-v16 were identified image segmentation and EGFP+ barcoded cells were classified as ‘neurons’, ‘oligodendrocytes’, ‘ambivalent’ based on the overlap with NeuN, Olig2, NeuN/Olig2, respectively. Barcoded cells were classified as ‘undetermined’ in absence of overlay with a cell type marker (n = 4 sections). **i, S**ummary of cell types amongst all EGFP+ barcoded cells. **j**, Targeted PCR on full length cDNA from gene expression libraries was used to achieve >4-fold enrichment of cloneIDs resulting in a total of 1,321 spots with ≥1 cloneID. **k**, Distribution of clone sizes for all reconstructed clones. **l, m**, All cloneIDs projected on reference representing non-immunostained (**l**) and immunostained (**m**) sections. **n**, Number of barcoded spots that contained a cell of the type ‘neuron’, ‘oligodendrocyte’, ‘undetermined’ or ‘ambivalent’.

Next, we developed Space-TREX, a method based on Spatial Transcriptomics (ST)^23^ that enables simultaneous clonal tracing and expression profiling of barcoded mouse brain sections in situ (Fig. 5a). We introduced immunostaining of intracellular antigens into the protocol, enabling combined profiling of spatial gene and protein expression together with clonal barcodes in the same tissue section (Fig. 5b and Extended Data Fig. 8a–d). Because ST relies on the capture of transcripts in spots with a diameter of 55 µm, most spots contain between one and ten cells with an average of about four cells (Extended Data Fig. 8e). However, not every cell in the tissue is barcoded, and, out of all spots containing an EGFP^+^ cell, 81% of spots contain only one barcoded cell, and the rest contain more than one barcoded cell (Extended Data Fig. 8f). Therefore, it can be assumed that a cloneID captured in a spot originates most often from a single barcoded cell, and we can reveal its identity using protein expression data collected for the same section.

We hybridized eight adjacent coronal sections from one postnatal day (P) 14 brain barcoded at E9.5 and used antibodies targeted to EGFP, NeuN and Olig2 to identify barcoded cells, neurons and oligodendrocytes, respectively (Fig. 5c). To establish a dataset containing information on spatial gene expression patterns, cell types, clones and neuroanatomical definitions, we aligned brain sections to the Allen Mouse Brain reference atlas using an integrated computational framework^39,40^ (Extended Data Fig. 8g–i). The entire dataset contained information on the transcriptional profiles of 28,746 spots that were distributed across all forebrain regions. We extracted a total of 1,321 cloneIDs, of which 1,079 cloneIDs were contained in 162 clones distributed across all brain regions (Fig. 5d,e and Extended Data Fig. 8j–n). The number of cells per clone in the Space-TREX data (6.7 ± 0.4, mean ± s.e.m., *n* = 162 clones; Fig. 5f) was significantly larger than the clone size observed in the TREX data (4.9 ± 0.3, mean ± s.e.m., *n* = 2,360 clones), indicating that cell loss leading to incomplete clones is reduced when using a spatial barcode readout.

In line with the TREX data, most clones showed a limited spread across all regions except for clones with cells located in white matter fiber tracts (Fig. 5g) that are known to be enriched for oligodendrocytes derived from highly migratory progenitors^32^. Although most clonally related cells crossed boundaries of major anatomical regions at low frequencies, intra-regional dispersion was more common, for example within cortical regions such as the amygdalar (AMY) and olfactory (OLF) areas as well as upper (UL) and deeper (DL) cortical layers (Fig. 5h). Interestingly, we observed extensive dispersion between either AMY/OLF or DL/UL, suggesting that most early progenitor cells are restricted to generate cell types of either area but undergo more widespread migration within each area.

**Fig. 6:**
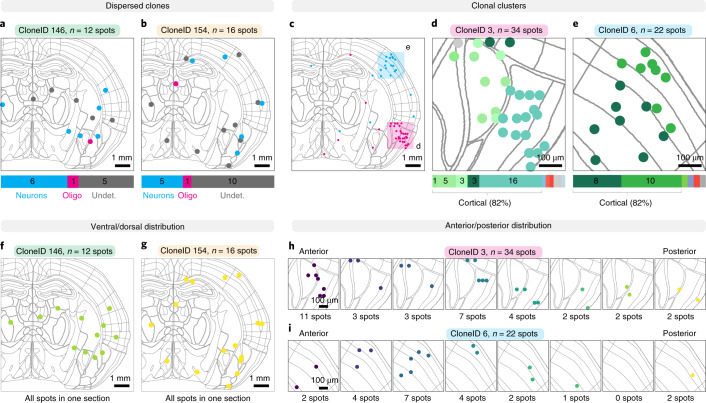
Dispersion and clustering of clonally related cells. Using Space-TREX of barcoded mouse brain sections, we recapitulated regional distributions of clonally related cells and uncovered specific distributions along the dorsoventral and anteroposterior axes. **a**,**b**, Tangential migration of progenitors leads to widespread dispersion of neurons and oligodendrocytes. Two clones are shown as examples, and clonally related spots are color-coded by cell type (blue, neuron; magenta; oligodendrocyte; gray, undetermined). Note that regional information was left out for clarity and can be found in Supplementary Fig. 12a,b. **c**–**e**, Radial migration of progenitors leads to clusters of clonally related cells illustrated with two clones (**c**) that mostly consist of cortical cells in the OLF areas (**d**) and the primary somatosensory area (**e**). Clonally related spots are color-coded by region, with cortical regions in different shades of green. Note that cell type information as well as detailed regional annotation were left out for clarity and can be found in Supplementary Fig. 12c-e. **f**,**g**, Cells of dispersed clones spread across the dorsoventral axis within the same 10-µm-thick section of the mouse brain. **h**,**i**, Cells from clustered clones spread from the most anterior to the most posterior brain section, spanning a region of 80 µm.

We used cell type information available for barcoded cells and found that clones containing both neurons and oligodendrocytes show an extensive spread across multiple regions (Fig. 6a,b and Supplementary Fig. 12a,b). This mode of dispersion likely corresponds to tangential migration well described for interneurons^41^ that also share a common early progenitor with oligodendrocytes, although these lineage relationships are not well understood^42^. We also observed neuronal clones that formed radially organized clusters mainly in the AMY/OLF areas as well as the UL/DL areas of the CX (Fig. 6c–e and Supplementary Fig. 12c–e). Although a few members of these clones were more widespread, more than 80% of all clonally related cells were found in larger clusters spanning areas of around 1.75 mm × 1.75 mm. Interestingly, cells from dispersed clones were distributed across the dorsoventral axis within a single 10-µm section, wherease cells from clustered clones were spread from the most anterior to the most posterior brain section, spanning 80 µm (Fig. 6f–i). Together, these data demonstrate that Space-TREX can be used for high-throughput mapping of clonal barcodes, gene expression and cell types in situ.

## Discussion

We developed TREX and Space-TREX for simultaneous clonal tracing and gene expression profiling of dissociated mouse brain cells and tissue sections, respectively. We found that the clonal dispersion across forebrain regions is limited, and only specific cell types are associated with dispersed clones. We discovered two fate-biased progenitor cell populations that exist as early as E9.5 in the hippocampal neuroepithelium, suggesting an unexpected early segregation of precursor cells. The clonal output of one progenitor population indicates that those cells are the origin for *Hopx*^+^ precursors that continue to become adult neural stem cells in the mouse dentate gyrus^33^.

We unraveled unique features of myeloid-derived clones, such as their large clone sizes and widespread dispersion across multiple forebrain regions compared to neuroectoderm-derived clones. The large clone size probably reflects the massive proliferation of brain macrophages required to colonize the entire CNS, because only a small number of precursors enter the brain before closure of the blood–brain barrier around E13, restricting access to immune cells that arise later in development^29^. Embryonic microglia migrate long distances within regions after entering the brain^43^, and we show that clonally related microglia also migrate extensively across anatomical boundaries to populate large areas of the brain. Microglia expansion and dispersion are central for brain homeostasis^44–46^ but remain only partially understood in particular at the clonal level. Thus, novel tools such as TREX enable systematic studies of the underlying molecular mechanisms within the context of microglia clonality.

Using Space-TREX, we provide, to our knolwedge, the first demonstration of high-density clonal tracking coupled to cell phenotyping and in situ sequencing of brain tissue. Compared to previous approaches that use complex in situ hybridization schemes and fluorescence microscopy for barcode detection^11,14^, Space-TREX relies on widely available reagents and DNA sequencing, thus enabling barcode readout in large tissue sections at scale^47^.

**Extended Data Fig. 9:**
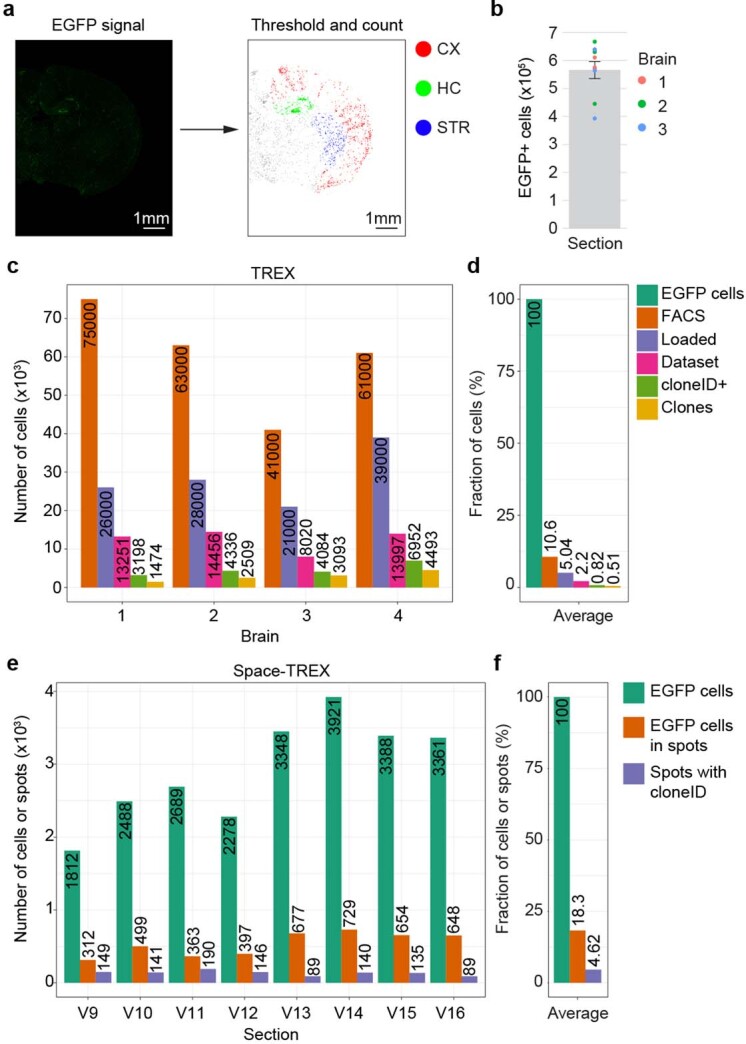
Cell loss and barcode dropouts. **a**, Workflow for counting the total number of barcoded EGFP+ cells in tissue sections. We counted EGFP+ cells separately in cortex (CX, red), hippocampus (HC, green) and striatum (STR, blue) in 50 µm thick coronal sections. For each region, we determined the labeling density (EGFP+ cells/mm^3^) and obtained the total number of cells by multiplying labeling density with volume estimations of the respective region. **b**, The average number of EGFP+ cells per brain is 566,000 ± 29,500 cells (n = 9 sections). Graph represents average number of cells per brain, error bars represent SD, colored dots represent individual data points corresponding to the number of labelled cells per section and brain. **c**, Recovery rate of labelled cells and barcodes at each step of the TREX protocol. Upon tissue dissociation isolated all EGFP+ cells contained in the suspension using bulk sorting (orange bar, ‘FACS’) and sorted cells were recovered via centrifugation. All recovered cells were transferred into the wells of a 10X Chromium chip (purple bar, ‘Loaded’) for droplet encapsulation. Following quality control and filtering, a subset of cells was contained in the final dataset (magenta bar, ‘Dataset’). A large fraction of cells had a barcode (lightgreen bar, ‘cloneID+’) of which many were contained in clones defined as a group of at least two cells sharing the same cloneID (yellow bar, ‘Clones’). **d**, Average recovery rate of cells and barcodes for all brains compared to the average total number of barcoded cells present in all three analyzed forebrain regions. **e**, Recovery rate of barcodes at each step of the Space-TREX protocol. Based on widefield images recorded for each section before library preparation, we could determine the total number of EGFP+ cells in a section (darkgreen bar, ‘EGFP cells’) and the number of those cells located in spots containing capture probes (orange bar, ‘EGFP cells in spots’). We displayed the final number of spots containing a cloneID (purple bar “Spots with cloneID”). **f**, Average recovery rate of cells and barcodes for all sections.

Currently, (Space-)TREX is limited by sparse sampling due to loss of barcoded cells after tissue dissociation (10.6% of cells recovered), isolation via fluorescence-activated cell sorting (FACS) (35–64% of sorted cells recovered) and droplet encapsulation (50% of loaded cells recovered) as well as cloneID dropout from a subset of sequenced cells (24–51% contain a cloneID), resulting in clonal information for about 0.51% of all barcoded cells initially present in the tissue (Extended Data Fig. 9). This would mean that the true clone size is 200-fold higher than the average clone size observed under our experimental conditions and that each neuroectoderm-derived clone contains about 800 cells on average, whereas each myeloid-derived clone is composed of 6,000 cells on average. Because a typical cortical clone labeled at E9.5 contains about 200 cells^48^, we consider our estimates an upper bound for true clone size.

**Extended Data Fig. 10:**
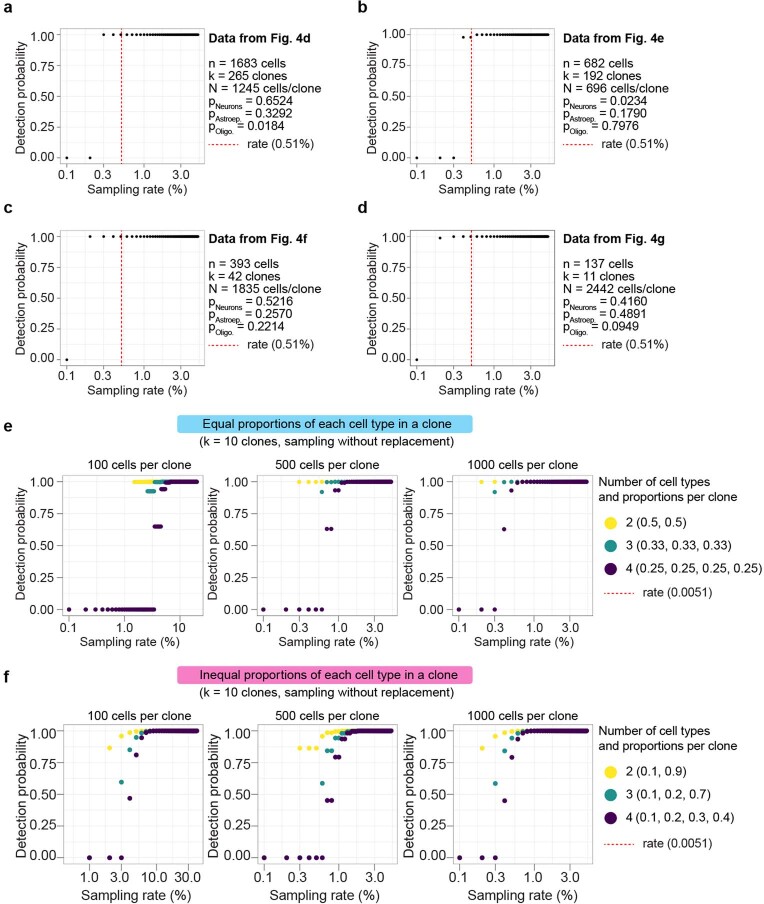
Detection probabilities of cell types in clones at low sampling rates. We determined the probability of detecting a cell type present in a clone with a certain proportion at low sampling rates (number of sequenced EGFP+ cells with clonal information compared to all EGFP+ cells present in tissue) using a multivariate hypergeometric distribution (see methods for details). **a-d**, The probability of detecting all three major cell types in our data is (close to) 100%. We used data from clones in the hippocampus (see Fig. 4d–g of this study) and estimated the true clone size N by first dividing the number of cells n by the number of clones k followed by multiplication with 1/rate where rate corresponds to the observed sampling rate of 0.51% (red line). We used the proportions for each major cell type (neurons, astroependymal cells, oligodendrocytes) and determined the probability of detecting the cell type with the lowest proportion when sampling k times without replacement. This analysis shows that even sparse sampling at a rate of 0.51% leads to 100% detection efficiency (except for dataset b with a detection efficiency of about 97.9%) under the described conditions. **e-f**, The probability of detecting a cell type in a clone is determined by clonal structure (number and proportion of cell types in a clone, clone size), cell sampling rate and the number of clones observed with a given structure. In general, lower sampling rates are sufficient to observe all cell types in larger clones with an equal cell type proportion (e) compared to smaller clones with unequal cell type proportion (f).

The observed cell and barcode recovery rates are in line with other approaches employing an scRNA-seq readout of genetic barcodes in various model systems and highlight a general challenge for such methods (Supplementary Table 1). Such approaches rely on sequencing a given clone with a specific clonal structure and size multiple times to provide statistically robust insights about the fate bias of progenitor cells. For example, we sampled clones from fate-biased progenitors in the HC 11–265 times, which is sufficient to also detect rare cell types with nearly 100% probability at the observed clonal sampling rate of 0.51% or less (Extended Data Fig. 10). Sparse sampling could be decreased by using a plate-based assay with higher RNA detection sensitivity^49^ or by employing a single-cell, high-sensitivity readout of cell types and barcodes using spatial transcriptomics.

Compared to classical fate mapping studies that rely on sparse labeling of cells in dozens to hundreds of (transgenic) animals, (Space-)TREX enables high-throughput dense reconstruction of clonal relationships using 10–30 times fewer animals (Supplementary Fig. 13). In contrast to CRISPR-based lineage tracing, our technology uses millions of diverse and compact barcodes that can be cloned as libraries, enabling straightforward barcode readout and clone reconstruction. Overall, we think that an integrated approach, such as Space-TREX, is needed to disentangle the complex relationships among cell identity, cell history and tissue anatomy that underlie the organisation of both the healthy and diseased brain.

## Methods

### Plasmid and lentivirus production

LV-EF1a-H2B-EGFP was constructed by exchanging the PGK1 promoter from LV-GFP^50^ with an EF1a promoter (Supplementary Table 2). Reporter constructs (Extended Data Fig. 4j) were cloned by exchanging EGFP with TagBFP (Evrogen), TagRFP (Evrogen) or emiRFP670 (ref. ^51^). The lentivirus plasmid library was generated by inserting an amplified oligonucleotide library (Supplementary Tables 2 and 3) into LV-EF1a-H2B-EGFP using Gibson assembly^52^ and transformation of electrocompetent Endura cells (Lucigen). Lentivirus particles (>10^9^ transducing units per milliliter) were generated by the core facility VirusTech at the Karolinska Institutet or by GEG-Tech.

### Sequencing of lentivirus preparations

Viral RNA was isolated using the NucleoSpin RNA Virus Mini Kit (Macherey-Nagel) and reverse transcribed using the SuperScript VILO cDNA Synthesis Kit (Invitrogen). The cDNA was used as template for cloneID amplification and indexing (Supplementary Tables 2 and 3). The resulting libraries were sequenced on an Illumina NextSeq (Supplementary Table 4), aligned against a reference containing the 30-bp cloneID and flanking regions using the BWA-MEM algorithm^53^. A custom BASH script was used to extract unique cloneIDs and corresponding read counts.

### Estimating the fraction of uniquely labeled cells

First, we calculated the total number of cells at the time point of injection. If *N*_*t*1_ is the number of labeled cells at E11.5, Δ*t* is the time difference in days and *f* is the frequency of cell divisions per day, then the number of transduced cells *N*_*t*0_ is:

$$N_{t1} = N_{t0} \ast 2^{{{{\mathrm{{\Delta}}}}}tf}\,{{{\mathrm{hence}}}}\,N_{t0} = \frac{{N_{t1}}}{{2^{{{{\mathrm{{\Delta}}}}}tf}}}$$

We determined $N_{t1} = 41,450$ cells, Δ*t* = 2 d and *f* = 2 divisions per day^54^, thus *N*_*t*1_ 2,591 ± 220 cells, or approximately 2,600, as noted in the main text.

Second, we estimated the fraction of uniquely labeled cells. For a number of uniformly distributed barcodes (*N*) and a small number of used barcodes (*k*) to label progenitor cells, the fraction *F* of uniquely labeled cells can be approximated as:

$$F = \left( {1-\frac{1}{N}} \right)^{k - 1}$$

However, the observed distribution of barcode abundance is not perfectly uniform in our library, which means that cells are more likely to be labeled with some barcodes than with others. The expected number^47,55^ of non-uniquely labeled cells, *E*(*X*), is then given by:

$$E\left( X \right) = k \ast \mathop {\sum}\limits_{i = 1}^N {p_i} (1 - (1 - p_i)^{k - 1})$$

where *p*_*i*_ is the probability of picking the cloneID *i* = 1…*N*, *k* is the number of infected progenitor cells and *N* is the total number of injected cloneIDs. We typically injected *N* = 0.94 × 10^6^ cloneIDs and estimated *k* = 2,591 cells, implying *E*(*X*) 11 non-uniquely labeled cells. This corresponds to 99.6% uniquely labeled cells, as stated in the main text.

### Mice

CD-1 mice (1× P11 female; 1× P11 male; 1× P12 male; 1× P12 female; 1× P14 male, 1× P14 female) obtained from Charles River Germany were used for all experiments. Animals were housed in standard housing conditions (ambient temperature of 20–22 °C and humidity of 40–60%), with a 12-h light/dark cycle with food and water ad libitum. All experimental procedures were approved by the Stockholms Norra Djurförsöksetiska Nämnd.

### Ultrasound-guided in utero microinjection

To target the developing mouse nervous system, a modified version of a published procedure^50^ was used. In brief, timed pregnancies were set up overnight, and plug-positive females were identified the next morning and counted as E0.5. Pregnant females at E9.5 of gestation were anaesthetized with isoflurane; uterine horns were exposed; each embryonic forebrain was injected with 0.6 µl of lentivirus; and 4–8 embryos were injected per litter. Surgical procedures were limited to 30 min to maximize survival rates.

### Immunostaining and imaging of embryonic and postnatal tissue

E11.5 mouse embryos were collected in ice-cold PBS, fixed in fresh 4% formaldehyde (FA) overnight at 4 °C, placed in 30% sucrose overnight at 4 °C, embedded in Tissue-Tek O.C.T. (Sakura) and cut into 20-µm-thick sections. Postnatal mice were sacrificed by isoflurane overdose, followed by transcardial perfusion with ice-cold PBS, followed by 4% FA. Brains were post-fixed in 4% FA overnight, and 50-µm sections were prepared using a VT1000S vibratome (Leica).

Sections were incubated with blocking/permeabilization buffer (5% donkey serum and 0.3% Triton X-100 in DPBS) and stained with antibodies against EGFP (chicken, 1:2,000, Aves Labs, AB_2307313), NeuN (rabbit, 1:500, Atlas Antibodies, AB_10602305), Sox9 (goat, 1:300, R&D Systems, AB_2194160), Sox10 (goat, 1:300, R&D Systems, AB_442208) or Iba1 (rabbit, 1:500, Wako, AB_839504) at 4 °C overnight. Sections were then washed with DPBS and incubated with fluorophore-conjugated secondary antibodies (donkey, 1:500, Jackson ImmunoResearch) against the respective species (anti-chicken Alexa Fluor 488, 703-545-155, AB_2340375; anti-rabbit Alexa Fluor 647, 711-605-152, AB_2492288; anti-goat Alexa Fluor 647, 705-605-147, AB_2340437) and DAPI (1 µg ml^−1^) in blocking buffer at room temperature for 1 h, followed by washing and mounting. Confocal images were captured with a laser scanning confocal microscope (LSM700, Carl Zeiss) using a Plan-Apochromat ×10/0.45 or ×20/0.8 objective. Image processing and analysis was performed using Fiji software^56^.

### Single-cell dissociations and flow cytometry

Mice were sacrificed with an overdose of isoflurane, followed by transcardial perfusion with ice-cold artificial cerebrospinal fluid (aCSF: 87 mM NaCl, 2.5 mM KCl, 1.25 mM NaH_2_PO_4_, 26 mM NaHCO_3_, 75 mM sucrose, 20 mM glucose, 2 mM CaCl_2_, 2 mM MgSO_4_). Mice were decapitated; the brain was collected in ice-cold aCSF; 1-mm coronal slices were collected using an acrylic brain matrix for mouse (World Precision Instruments); and the regions of interest were microdissected under a stereo microscope with a cooled platform. Tissue pieces were dissociated using the Papain dissociation system (Worthington Biochemical) with an enzymatic digestion step of 20–30 min, followed by manual trituration using fire-polished Pasteur pipettes. Dissociated tissue pieces were filtered through a sterile 30-µm aCSF-equilibrated Filcon strainer (BD Biosciences) into a 15-ml centrifuge tube containing 9 ml of aCSF and 0.5% BSA. The suspension was mixed well; cells were pelleted in a cooled centrifuge at 300*g* for 5 min; supernatant was carefully removed; and cells were resuspended in 1 ml of aCSF containing reconstituted ovomucoid protease inhibitor with BSA. A discontinuous density gradient was prepared by carefully overlaying 2 ml of undiluted albumin inhibitor solution with 1 ml of cell suspension, followed by centrifugation at 100*g* for 6 min at 4 °C. The supernatant was carefully removed; the cell pellet was resuspended in 1 ml of aCSF containing 0.5% BSA; and the cell suspension was transferred to a round-bottom tube (BD Biosciences) for flow cytometry. Single EGFP^+^ cells were sorted on a BD Influx equipped with a 140-µm nozzle and a cooling unit with a sample temperature of 4 °C and collected into a DNA LoBind tube (Eppendorf) containing aCSF with 0.5% BSA. All EGFP^+^ cells per sample were sorted and pelleted in a cooled centrifuge at 300*g* for 5 min. The supernatant was carefully removed; the cell pellet was resuspended in a minimal volume of aCSF; and the cell concentration was determined using a Bürker chamber. Importantly, aCSF equilibrated in 95% O_2_/5% CO_2_ was used in all steps, and cells were always kept on ice or at 4 °C except for enzymatic digestion.

### scRNA-seq

Two brains (brains 1–2) were processed using the 10x Genomics Chromium Single Cell Kit Version 2 (v2), and three brains (brains 3–5) were processed using the 10x Genomics Chromium Single Cell Kit Version 3 (v3) (Supplementary Table 4). Suspensions from barcoded brains were prepared as described above, counted and resuspended aCSF and added to 10x Chromium RT mix. Suspensions from control brains were prepared as described above, diluted in aCSF to concentrations between 800 and 1,000 cells per microliter and added to 10x Chromium RT mix. For downstream cDNA synthesis (12 polymerase chain reaction (PCR) cycles), library preparation and sequencing, we followed the manufacturer’s instructions.

### Data normalization and cell filtering for scRNA-seq

Overall, three regions from four barcoded brains and from one control brain were sequenced using 10x Chromium v2 or v3. Because the number of cells per region for the control brain was much higher than the corresponding number of cells for any barcoded brain (Supplementary Table 4), we downsampled the control datasets to about 9,000 cells (CX), 8,000 cells (HC) and 7,000 cells (STR). The gene expression matrices obtained after running Cell Ranger count were merged by region (CX, STR and HC) using merge() in Seurat version 3 (ref. ^57^). All genes expressed in ~0.1% of all cells were kept, and all cells expressing 500–10,000 genes were kept in the merged data. The data were log-normalized with a scale factor of 10,000 using the NormalizeData() function, followed by linear transformation (scaling) of data. Doublet removal was done using mutually exclusive markers for various cell types (*Igf2, Pf4, Hexb, Rsph1, Pdgfra, Bmp4, Mog, Clic6, Rgs5, Cldn5, Reln, Igfbpl1, Slc32a1, Slc17a7* and *Aldoc*). A cell cycle score was assigned to each cell, and the difference between the G2M and S phase scores was regressed out. Highly variable features were selected using FindVariableFeatures(), followed by principal component analysis and the use of significant principal components (between 10 and 30) for graph-based clustering (shared nearest neighbor graph calculation and clustering using Louvain). After determining differentially expressed genes, we manually assigned major cell classes to each cluster (astroependymal, immune, neurons, oligodendrocytes and vascular) using canonical markers. We then split cells by major cell type, performed subclustering and extensively annotated each cluster based on canonical marker genes from published data and from www.mousebrain.org. At each step, we removed (1) clusters classified with ambiguous labels and (2) outlier cells on the fringes of clusters in uniform manifold approximation and projection (UMAP) space to further eliminate doublets. We merged all cells into a single file together with metadata and annotations. The filtered cellIDs were exported and used as input for cloneID extraction and clone calling.

### Biological pathway analysis between barcoded and control samples

To investigate the effect of lentivirus transduction on cellular physiology, we analyzed 195 genes expressed during virus infection (KEGG pathway: mmu05170). We downsampled the dataset such that each cell type per condition contained an equal number of cells. We plotted expression values of non-zero expressed genes related to virus infection for single cells as heat maps grouped by condition or major cell type. For each cell type, we analyzed differentially expressed genes between both conditions (logfc.threshold ≥ 1) on normalized and variance-stabilized downsampled datasets.

### CloneID enrichment from cDNA

A nested PCR strategy was employed for enrichment of cloneIDs from full-length cDNA (Supplementary Tables 2 and 3). Each amplicon library was sequenced on a MiSeq or NovaSeq 6000 (Supplementary Table 4). We used Cell Ranger version 3.0.1 count for data processing of amplicon libraries and the TREX pipeline (see below) to extract cloneIDs.

### Extraction of cloneIDs and clone calling for scRNA-seq

Raw 10x Genomics Chromium v2 or v3 sequencing data were pre-processed with Cell Ranger version 3.0.1. As reference for read mapping, Cell Ranger was configured to use a custom reference consisting of the GRCm38 (mm10) genome and an additional sequence representing the H2B-EGFP-N transgene, in which the cloneID region was marked with ‘N’ wildcard characters. The resulting BAM file of aligned sequencing reads was then processed with TREX, our custom Python tool for cloneID extraction and clone calling. TREX uses only reads from filtered cells (see above) that align to the H2B-EGFP-N transgene. CloneIDs are recovered from those alignments that cover the masked cloneID region. If soft clipping is encountered at one of the bases adjacent to the region, the alignment is assumed to continue ungapped into the region. All cloneIDs with identical unique molecular identifiers (UMIs) that come from the same cell (have the same cellID) are collapsed to a consensus sequence. To error-correct cloneIDs, they are single-linkage clustered using a Hamming distance of at most 5 as linking criterion. In each cluster, all of its cloneIDs are replaced with the cloneID occurring most frequently in that cluster. From the resulting final cellID–cloneID combinations, those that are supported by only one UMI and one read are discarded. Also removed are cloneIDs that are supported by only one UMI and have a high frequency in another cell. We assume that those cloneIDs are contaminations.

The cleaned data are transformed into a count matrix showing UMI counts for each cloneID in each cell. This matrix is used to sort cells into clones of cells with similar cloneID combinations. In brief, the Jaccard similarity between each pair of cloneID-expressing cells was calculated using the R package proxy^58^. A Jaccard score of 0.7 was used as a cutoff for related cells^15^, and clones were defined as groups of two or more related cells.

### Calculation of clonal coupling scores

For each brain, we calculated clonal coupling scores^20^ considering all clones containing at least three cells per clone. We randomized the clone–cell type associations, while preserving the number of cell types related to each clone and the number of clones related to each cell type, to create 1,000 randomized datasets^59^. We compared the observed clonal data to randomized datasets to obtain empirical *P* values and *z*-scores indicating, for each pair of cell types, how often we expect to see the observed clonal association. To summarize the clonal coupling scores for four brains, we kept only cell types found in clones in all brains. For each brain, the Pearson correlations of *z*-scores between each pair of cell types were calculated, and the correlation coefficients were transformed using Fisher *z*-transformation and averaged to represent clonal coupling scores for all brains.

### Calculation of detection probabilities at low sampling rates

We estimated the probability of sampling (without replacement) at least one cell from each cell type in each clone using a multivariate hypergeometric distribution implemented in the function dmvhyper from extraDistr^60^. Given the number of cells from different cell types present in a clone and the sampling rate, we can calculate

$$P_{detect\_all}\left( {cell\_type\_distr,s} \right) = \mathop {\sum }\limits_{x{\it{\epsilon }}X}^{sampled\_cells} PMF_{mv\_hyper}\left( {x,cell\_type\_distr,s} \right)$$

where

- *cell_type_distr* is the number of cells from each cell type in the clone.
- *s* is the number of sampled cells.
- *X* is all possible combinations of *s* sampled cells for the given clone, so that we have at least one cell from each cell type.
- *PMF*_*mv_hyper*_ is the probability mass function for the multivariate hypergeometric distribution.

Next, assume that we have *N* similar clones with the same number of cells distributed over the same cell types as above. If we sample cells from all the *N* clones, the probability of sampling at least one cell from each cell type in at least one clone is given by the binomial distribution:

$$\mathop {\sum }\limits_{n = 1}^N PMF_{binom}\left( {n,N,P_{detect\_all}\left( {cell\_type\_distr,s} \right)} \right)$$

### Tissue processing and library preparation for ST

Mice were sacrificed. Then, brains were collected in ice-cold aCSF, transferred to ice-cold Tissue-Tek O.C.T. (Sakura) and snap-frozen at −40 °C in a bath of isopentane and dry ice. Eight consecutive 10-µm sections around AP −1.65 mm from bregma were collected for processing using the 10x Genomics Visium Spatial Gene Expression Kit.

The first four sections (V9–V12) were fixed in ice-cold methanol, followed by rapid imaging (<15 min for all sections) of EGFP and transmitted light signal using an epifluorescence microscope (Axio Imager.Z2, Carl Zeiss) equipped with a Plan-Neofluar ×10/0.3 M27 objective before further processing following the manufacturer’s instructions. The remaining four sections (V13–V16) were fixed in ice-cold methanol, briefly rinsed with DPBS, incubated with DPBS containing DAPI (1 µg ml^−1^), FluoTag-X4 anti-GFP conjugated to Atto488 (1:200, Nanotag Biotechnologies), NeuN-Alexa 568 (rabbit, 1:400, Abcam, ab207282), Olig2-Alexa 647 (rabbit, 1:200, Abcam, ab225100) and RNaseOUT (1 U µl^−1^) at room temperature for 10 min. The sections were washed two times for 1 min with DPBS containing RNaseOUT (1 U µl^−1^) and mounted in 85% glycerol containing RNaseOUT (1 U µl^−1^), and images were captured for all four fluorescent channels as well as the transmitted light channel. The coverslip was removed by immersing the slide in water. Then, the slide was dried for 5 min at 37 °C and further processed following the manufacturer’s instructions, starting with the tissue permeabilization step.

### Data and image analysis for ST

For each section, the registered microscope image was used for manual alignment and tissue detection using the Visium Manual Alignment Wizard (10x Genomics), followed by running Space Ranger version 1.0.0 (Supplementary Table 4). Each dataset was separately processed in Seurat version 3 (ref. ^57^), and only spots that expressed at least 300 genes were kept. We used SCTransform^61^ for data processing, merged datasets and exported spot IDs as input for cloneID extraction and clone calling.

Fluorescent images acquired for four sections (V13–V16) were processed in R using a custom segmentation workflow that entails (1) two-dimensional (2D) fast Fourier transform convolution filtering, (2) image correction, (3) thresholding, (4) removal of speckles or other abnormal shapes and (5) watershedding to identify and label cells. The segmentation workflow was applied to each of three channels: EGFP (barcoded cells), NeuN (neurons) and Olig2 (oligodendrocytes). To find co-localizing signals across two channels, A and B, an overlap score was estimated for all pairs of nuclei (*i*, *j*) as intersect(A_i_, B_j_)/min(A_i_, B_j_) where A_i_ and B_j_ are the sets of pixels defining nuclei *i* and *j*. An overlap score of at least 50% was used to determine if the signal originated from the same nuclei. For alignment of all four sections, we used a manual image registration method implemented in the ManualAlignImages function from the STUtility package^62^. The raw NeuN images were masked, and tissue edges were manually rotated or shifted to fit the image and spot coordinates of images V14–V16 to the reference image V13. All capture spot coordinates from V14–V16 were transformed to align with the coordinate system of V13 using the learned transformation functions. The same transformations were applied to the coordinates of the previously segmented nuclei in V14–V16. We calculated the pairwise 2D Euclidean distances between aligned spots and nuclei and selected the cell with shortest distance to the centroid position of each cloneID^+^ spot for assignment of cell type identity. For alignment of all four H&E-stained sections (V9–V12), we used an automated image registration method implemented in the AlignImages function from STUtility with image V9 as reference. All capture spot coordinates from V10–V12 were transformed to align with the coordinate system of V9, using the learned transformation function.

Registration of aligned images of brain tissue sections to the standardized Allen Mouse Brain Atlas was done using WholeBrain^40^. We used an extended and inverted version of the H&E target image V9 and the NeuN target image V13 with bregma coordinates AP −1.65 mm for registration of an entire brain section to the Allen Mouse Brain Atlas.

### Statistics and reproducibility

No statistical method was used to pre-determine sample size, but our sample sizes match typical numbers used in scRNA-seq and ST experiments^2,6,23,63,64^. For TREX, one male and one female EGFP^+^ animal were randomly selected from two different litters, and the control mouse was randomly selected from a third litter. For Space-TREX, one EGFP^+^ mouse was randomly selected from a pool of littermates. For TREX, we collected all EGFP^+^ cells per brain region to sample the maximum amount of clonally related cells and to have enough cells for each cell type, allowing further quantitative analysis. Data distribution was assumed to be normal, but this was not formally tested. No data were excluded from the analyses. The allocation to experimental groups (barcoded versus control) could not be randomized because it was necessary to specifically isolate EGFP^+^ cells present only in barcoded brains. Blinding was not applicable because control and barcoded samples were of similar age, differentiable based on EGFP fluorescence, and our results were based on analysis of clonal barcodes present only in EGFP^+^ cells.

### Reporting Summary

Further information on research design is available in the Nature Research Reporting Summary linked to this article.

## Online content

Any methods, additional references, Nature Research reporting summaries, source data, extended data, supplementary information, acknowledgements, peer review information; details of author contributions and competing interests; and statements of data and code availability are available at 10.1038/s41593-022-01011-x.

## Supplementary information

Supplementary Information Supplementary Figs. 1–13

Reporting Summary

Supplementary Table 1 Recovery rates and clone sizes for combined profiling of lineages and cell types using scRNA-seq in mammalian model systems. Compared to five other recent studies in mammals, our approach yields similar barcode recovery rates and numbers of cells per clone and, to our knowledge, is the only method that has been extensively characterized to meet the needs for studying neurogenesis in vivo. Note that we restricted this analysis to recent papers where the relevant information was accessible.

Supplementary Table 2 Sequences of oligonucleotides used for plasmid cloning and cloneID library preparations.

Supplementary Table 3 PCR protocols used for preparing cloneID sequencing libraries.

Supplementary Table 4 Overview of cloneID, single-cell and ST next-generation sequencing libraries.

## Data Availability

All RNA sequencing datasets generated in this study are deposited in the Gene Expression Omnibus under accession code GSE153424. All processed single-cell and spatial transcriptomics datasets are available as RDS files using the following link: https://kise-my.sharepoint.com/:f:/g/personal/michael_ratz_ki_se/EndBZ9VI_rRHmHzZxrAwSZQBeE9e4RNmktbuCcHir1a5qQ?e=Ge2Fqm, with password 8RMG.xbzH?3v9Ef4. Source data are provided with this paper.

## Source data

Source Data Fig. 1 Statistical Source Data
Source Data Fig. 2 Statistical Source Data
Source Data Fig. 3 Statistical Source Data
Source Data Fig. 4 Statistical Source Data
Source Data Fig. 5 Statistical Source Data

## Code availability

TREX source code is available under the MIT license from https://github.com/frisen-lab/TREX. Image segmentation and alignment code is available under the MIT license from https://github.com/ludvigla/TREXSeg.
