# Supplementary material for: Clonal relations in the mouse brain revealed by single-cell and spatial transcriptomics
Source: Nat Neurosci. 2022 Feb 24;25(3):285–94. doi: 10.1038/s41593-022-01011-x (PMC8904259; doi:10.1038/s41593-022-01011-x)
Supplement: Supplementary file 1 — Supplementary Figs. 1–13 [file 41593_2022_1011_MOESM1_ESM.pdf]

nature portfolio

https://doi.org/10.1038/s41593-022-01011-x

Supplementary information

Clonal relations in the mouse brain revealed by single-cell and spatial transcriptomics

In the format provided by the authors and unedited

Supplementary information

Clonal relations in the mouse brain revealed by single-cell and spatial transcriptomics

Supplementary figures

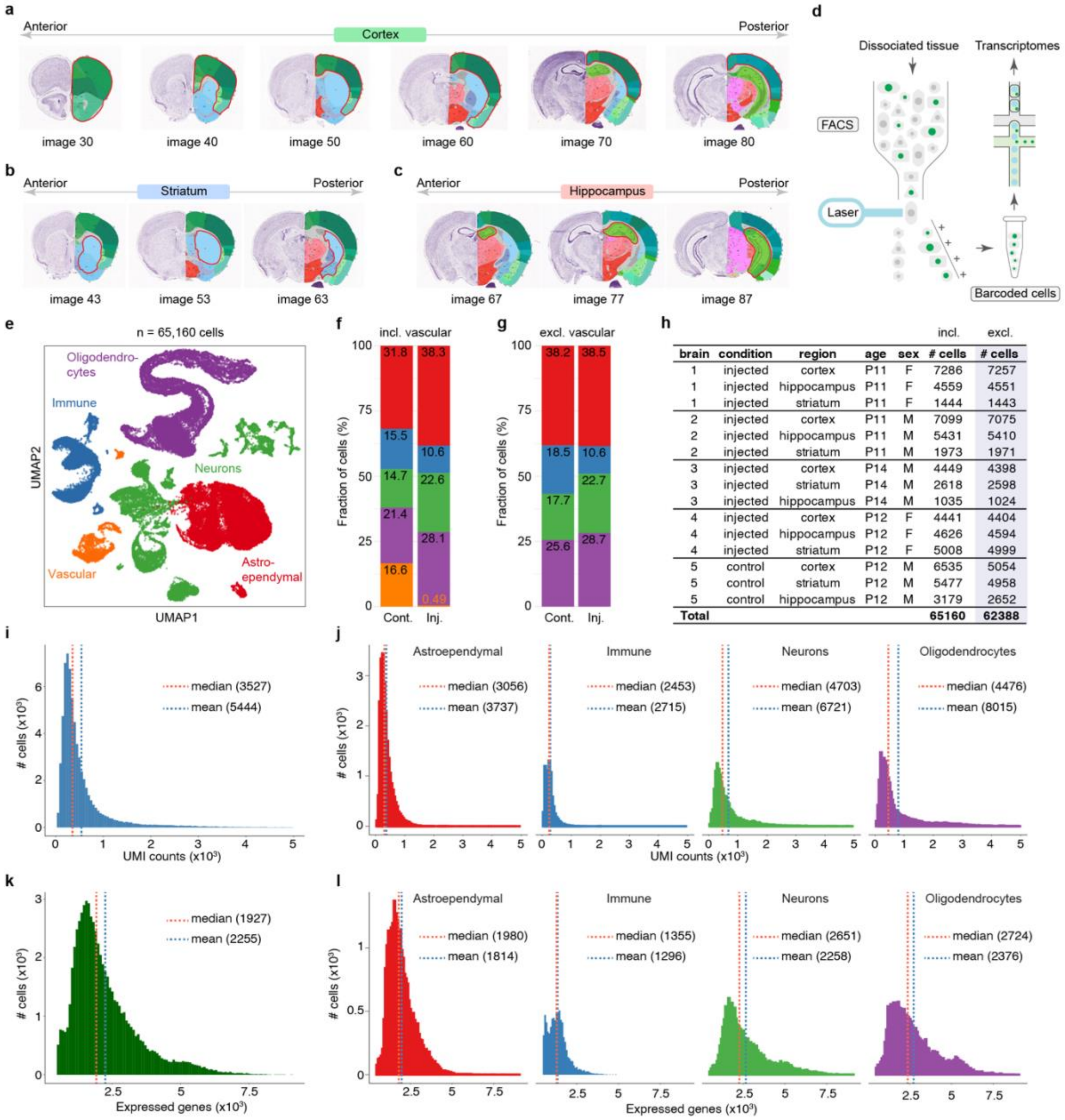

brain	condition	region	age	sex	incl. # cells	excl. # cells
1	injected	cortex	P11	F	7286	7257
1	injected	hippocampus	P11	F	4559	4551
1	injected	striatum	P11	F	1444	1443
2	injected	cortex	P11	M	7099	7075
2	injected	hippocampus	P11	M	5431	5410
2	injected	striatum	P11	M	1973	1971
3	injected	cortex	P14	M	4449	4398
3	injected	striatum	P14	M	2618	2598
3	injected	hippocampus	P14	M	1035	1024
4	injected	cortex	P12	F	4441	4404
4	injected	hippocampus	P12	F	4626	4594
4	injected	striatum	P12	F	5008	4999
5	control	cortex	P12	M	6535	5054
5	control	striatum	P12	M	5477	4958
5	control	hippocampus	P12	M	3179	2652
**Total**					**65160**	**62388**

Supplementary Figure 1. Experimental design, cell numbers and gene expression metrics.

a-c, Each brain was cut using a 1 mm coronal brain slicer and brain regions shown in red contour along the anterior/posterior axis were dissected for tissue dissociation. Image numbers refer to the image number from the Allen Brain Atlas (http://atlas.brain-map.org/atlas?atlas=1#atlas=1). **a**, Cortex samples included the cortical plate (except for hippocampal formation) and most parts of the cortical subplate. **b**, Striatum samples included the caudoputamen and nucleus accumbens. **c**, Hippocampus samples contained the hippocampal formation (except for the entorhinal area). **d**, For each postnatal brain, dissected regions were dissociated separately and barcoded cells were isolated using fluorescence-activated cell sorting (FACS). We used droplet microfluidics (10X Genomics Chromium)

to reveal the transcriptomes of barcoded cells. **e**, Initial clustering of cells from both barcoded and non-injected control brains revealed five major cell types annotated and visualized in a UMAP. **f**, **g**, Compared to non-injected control samples (Cont.), barcoded brains (inj.) contained very few vascular cells, because few blood vessels are present at the time of injection (**f**). Therefore, we removed vascular cells from all datasets (**g**). **h**, Table summarizing final cell numbers for each region and replicate. **i, j**, Number of transcripts (unique molecular identifiers, UMIs) per cell for the entire dataset (**i**) and for each major cell type (**j**). **k, l**, Number of expressed genes per cell for the entire dataset (**k**) and for each major cell type (**l**).

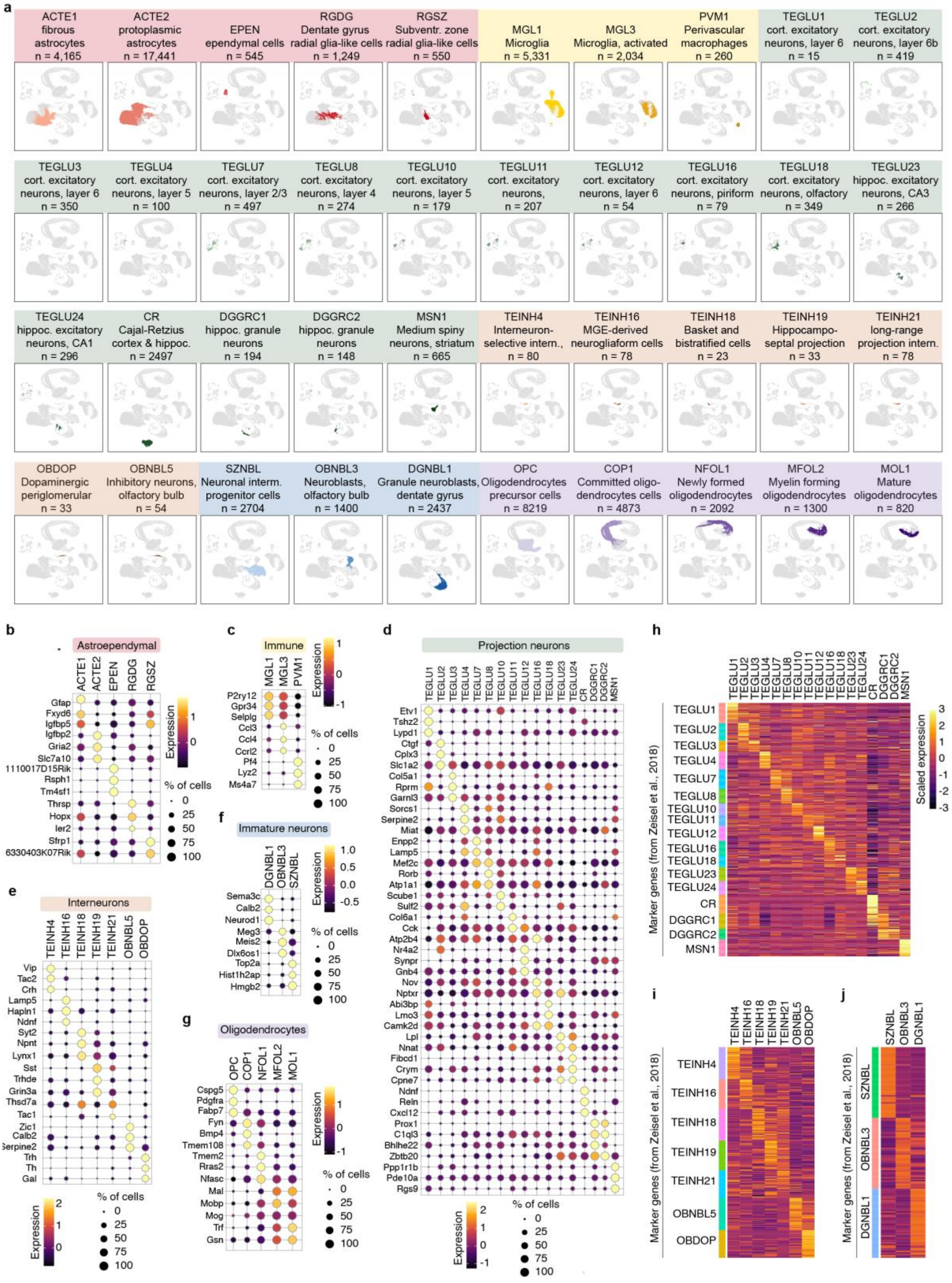

Supplementary Figure 2. Cell types and marker genes.

a, Separate UMAP visualizations for all cell types and corresponding number of cells per type identified in this study. Colors indicate six broader cell type classes: astroependymal (reds), immune (yellows), interneurons (oranges), projection neurons (greens), immature neurons (blues) and oligodendrocytes

(purples). **b-g**, Gene expression of markers for each cell type belonging to the six broad cell type classes astroependymal (b), immune (c), projection neurons (d), interneurons (e), immature neurons (f) and oligodendrocytes (g). For each cell type the top three marker genes were identified, and unique genes plotted as dot plots. Expression values represent scaled average gene expression per cell type. **h-j**, We used the same mnemonic identifiers from a previous mouse brain atlas (www.mousebrain.org) to annotate cell types found in our study. Each heatmap shows the expression for the unique differentially expressed genes (rows) from the mouse brain atlas for the corresponding cluster (columns) from this study for projection neurons (**h**), interneurons (**i**) and immature neurons (**j**).

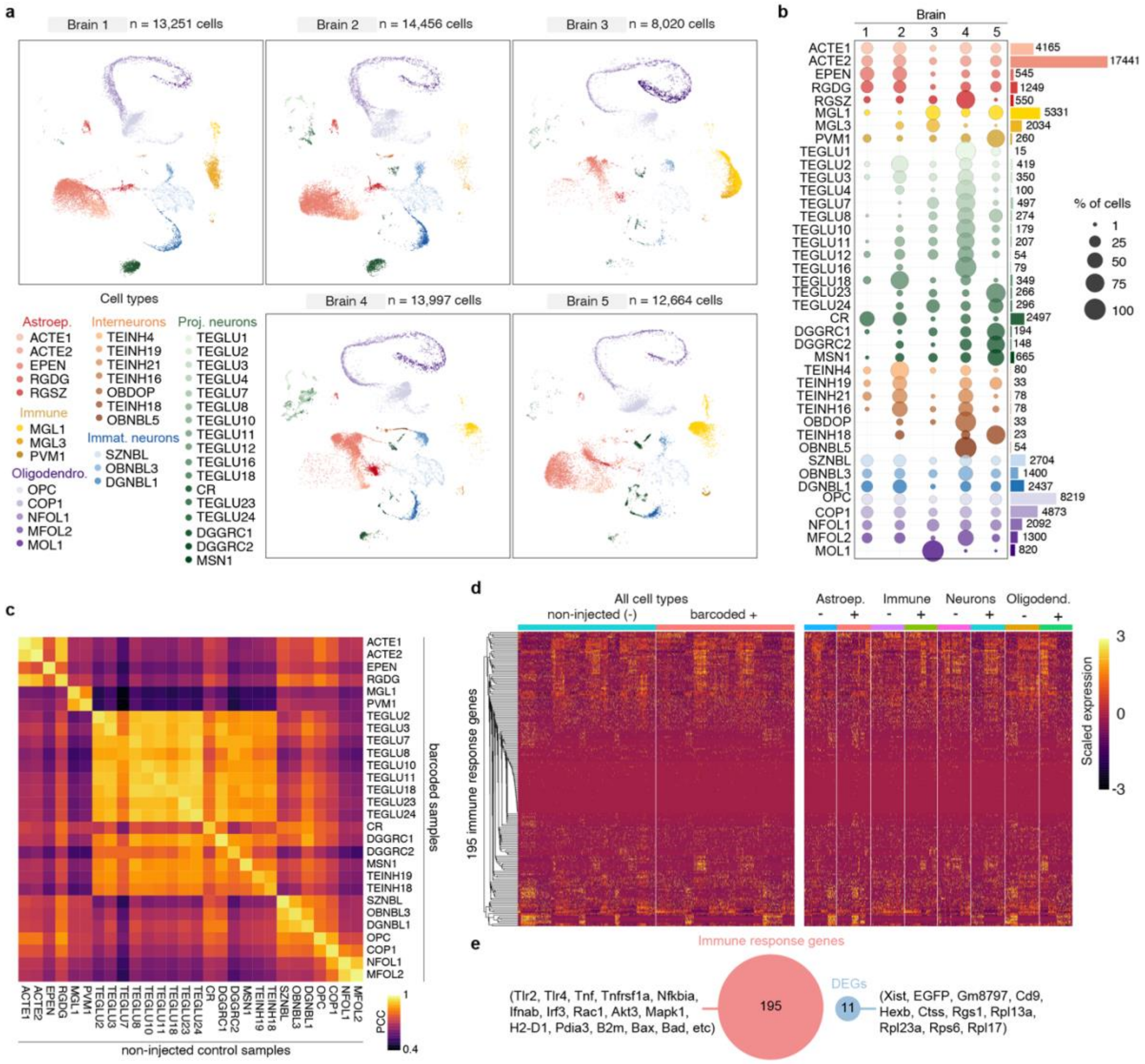

Supplementary Figure 3. Lentiviral barcoding does not perturb cell physiology.

a, UMAP visualizations for all 40 cell types split by biological replicate (brain). **b**, Barcoded cells (from brains 1-4) and control cells (from brain 5) are distributed relatively evenly across each cell type. Some variability is expected due to variations in progenitor labeling, cell type sampling and slight age differences between mice used. Dot plot shows fraction of cells per cell type across all brains. Bar plot shows total number of cells per cell type. **c**, Gene expression is similar between control and barcoded cells. Heatmap showing Pearson correlation coefficients (PCC) between average gene expression values for each cell type from barcoded and control brains. A high PCC value indicates similar gene expression patterns between both conditions. Clusters containing at least 5 cells per condition and cell type were analyzed. **d,** Immune response genes are not upregulated in barcoded cells. Heatmap displaying gene expression for 195 immune response genes (KEGG pathway "Human immunodeficiency virus 1 infection", mmu05170) for an equal number of single cells grouped by condition (left) or grouped by cell type and condition (right). **e**, Only few genes are differentially expressed between barcoded and control cells and do not overlap with immune response genes. Venn

diagram showing all differentially expressed genes between barcoded and control cells for each cell type and their non-overlap with immune response genes.

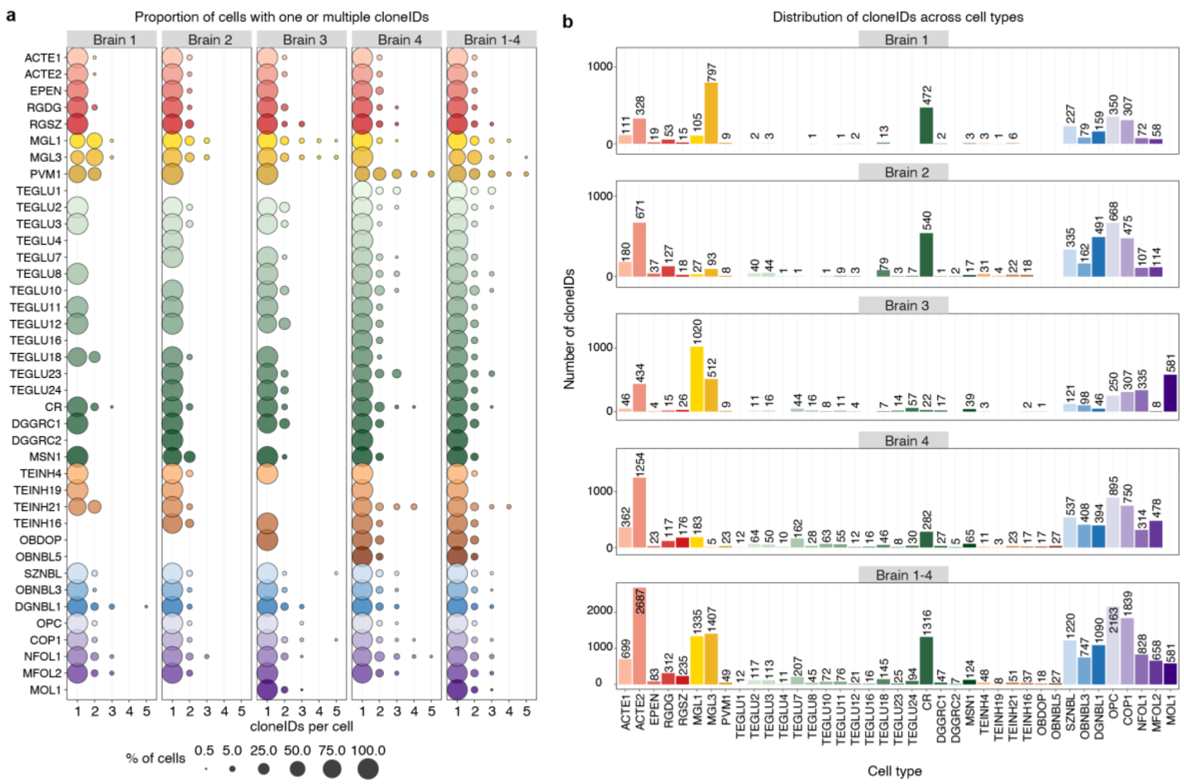

Supplementary Figure 4. Distribution of cloneIDs across cell types and brains.

a, Proportion of cells expressing one or multiple cloneIDs. Dot plots show that most cell types express only one cloneID while some cell types express multiple cloneIDs across barcoded brains. **b**, Bar plots showing that the total number of cloneID positive cells varies among cell types and across brains.

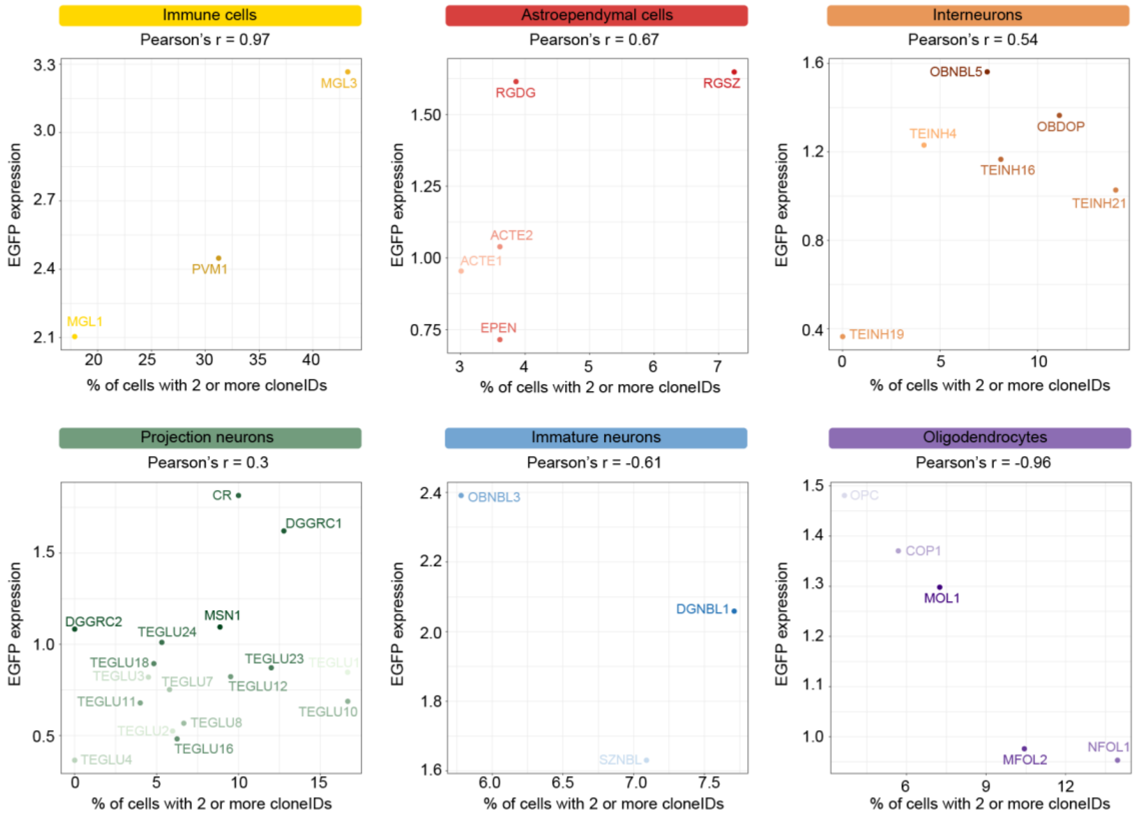

Supplementary Figure 5. Correlation between fraction of cells with multiple cloneIDs and cloneID expression levels.

Scatter plots showing the relation between average EGFP expression level (y-axis) and fraction (%) of cells with 2 or more cloneIDs (x-axis) for each identified cell subtype grouped by major cell class ordered by decreasing Pearson correlation coefficients (r). Correlation coefficients vary greatly depending on cell type and indicate strong positive correlation (immune cells), moderate positive correlation (astroependymal cells, interneurons), weak positive correlation (projection neurons), moderate negative correlation (immature neurons) and strong negative correlation (oligodendrocytes) between EGFP-cloneID expression levels and fraction of cells with 2 or more cloneIDs.

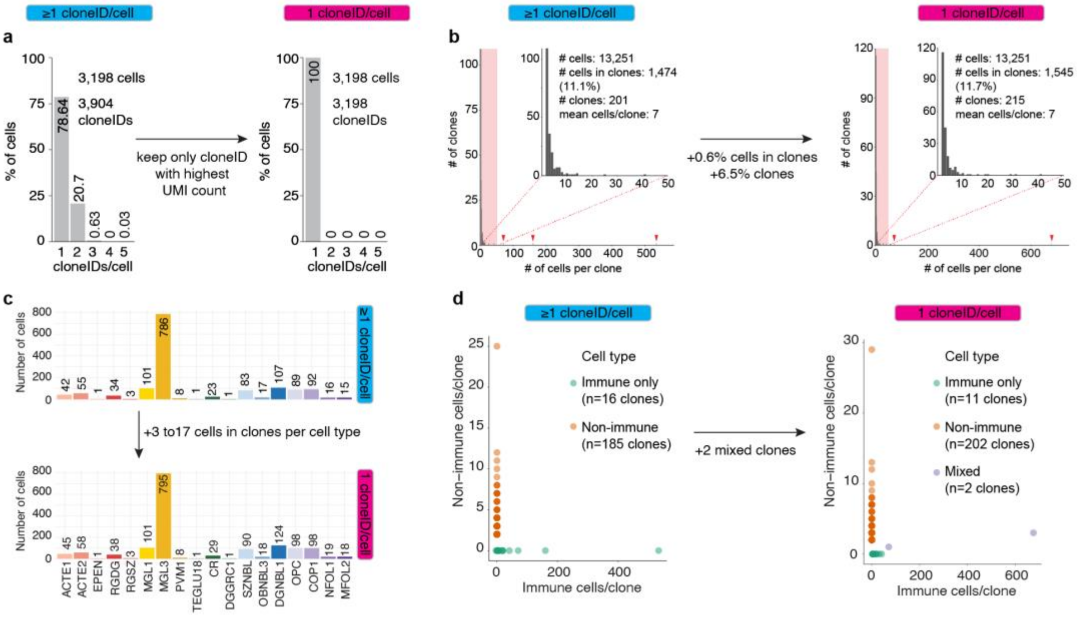

Supplementary Figure 6. Removal of multiple cloneIDs from cells leads to higher error in clone reconstruction.

a, When two or more cloneIDs were found in the same barcoded cell of brain 1, we only kept the cloneID with the highest UMI count and removed all other cloneIDs. **b,** Clone size histogram showing that 0.6% more cells are contained in clones and 6.5% more clones are reconstructed when using only a single cloneID. **c,** Depending on cell type, 3 to 17 more cells were found in clones when using only a single cloneID. **d,** Cell types that often express more than one cloneID such as immune cell clones are affected by “lumping” errors leading to less clones with a larger size and a higher number of incorrectly associated neuroectoderm-derived cells.

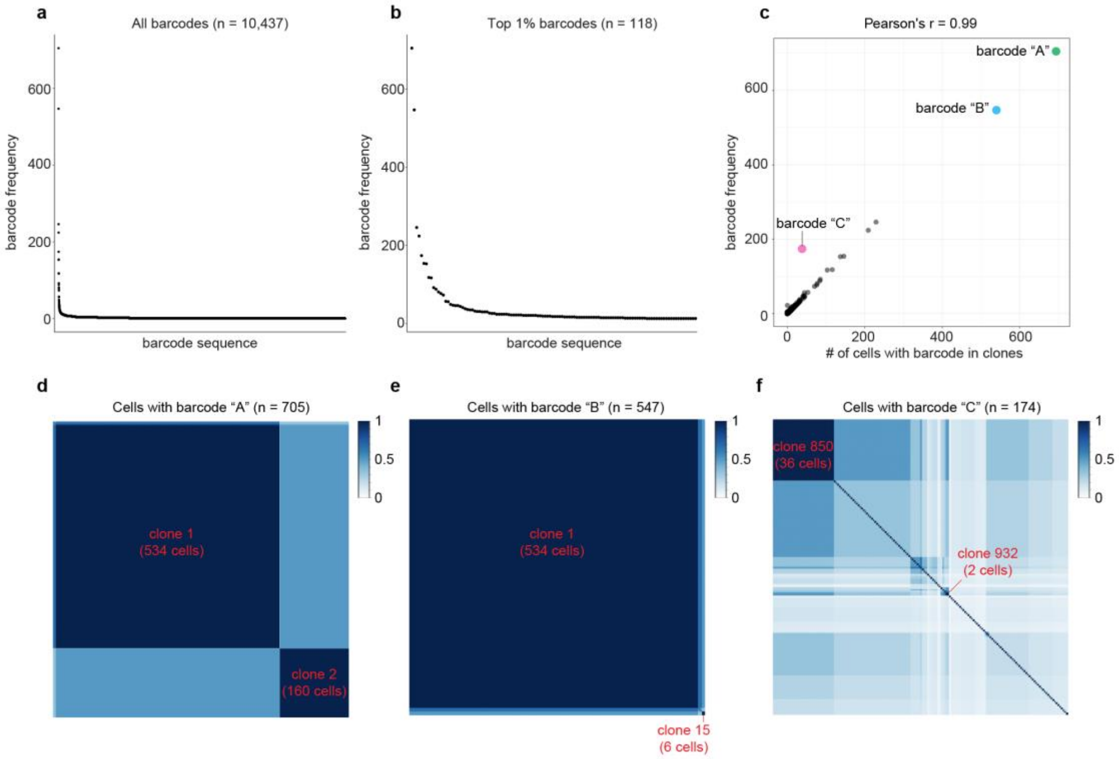

Supplementary Figure 7. Frequency distribution of genetic barcodes.

a, b, Rank plots of all unique barcode sequences (a) and the top 1% barcode sequences (b). **c,** A nearly perfect Pearson correlation (r = 0.99) was observed between the number of total occurrences for each barcode sequence (frequency) and the number of cells with a given barcode in clones. Three barcodes named "A", "B" and "C" (nucleotide sequences were omitted for clarity) were highlighted to illustrate the relationships between barcode frequency and number of cells with barcodes in clones in more detail in the panels below. **d-f,** Correlation plots showing Jaccard similarities between all possible pairs of cells expressing three selected barcodes. The two most abundant barcodes "A" and "B" are distributed across three clones (d, e). Clone 1 is defined by the co-expression of both barcodes "A" and "B" and contains 534 cells. Clone 2 expresses only barcode "A" and contains 160 cells. Clone 15 expresses only barcode "B" and contains 6 cells. Barcode "A" is distributed across a total of 705 cells of which the majority (n = 694 cells) is contained in clones while barcode "B" was found in a total of 547 cells of which the majority (n = 540 cells) was detected in clones. The fifth most abundant barcode "C" was found in clone 850 (n = 36 cells) and in clone 932 (n = 2 cells) which co-expresses a second barcode and is therefore considered a separate clone. Out of 174 cells in total expressing barcode "C", only 38 cells are contained in clones. We note that barcode "C" is an outlier barcode since it is the only barcode with a high frequency and low number of cells in clones.

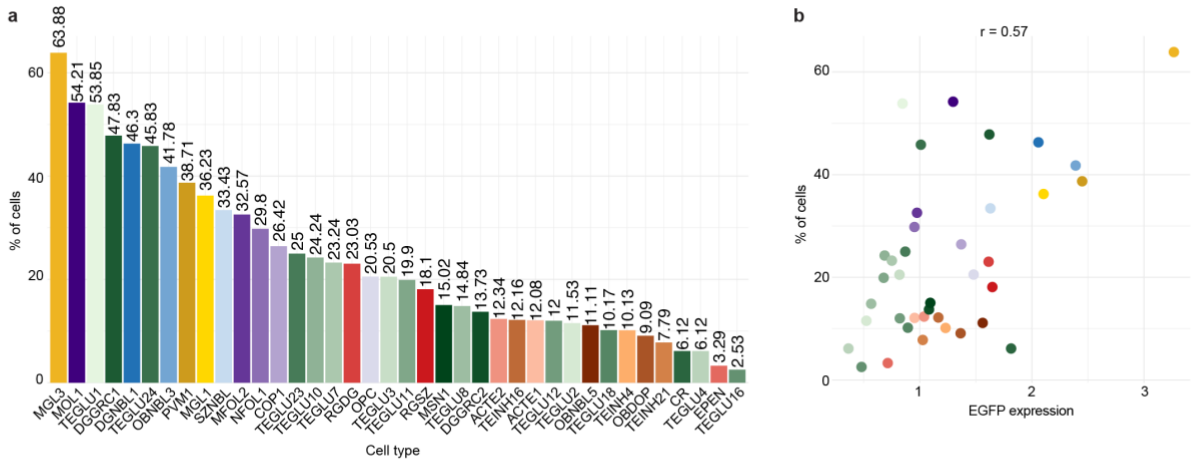

Supplementary Figure 8. Number of cells in clones correlates with EGFP expression levels in each cell type.

a, Bar plot showing the proportion of cells in clones for each cell type ordered by decreasing proportion. **b**, Scatter plot showing a high correlation (Pearson's r = 0.57) between proportion of cells and average normalized EGFP expression for each cell type.

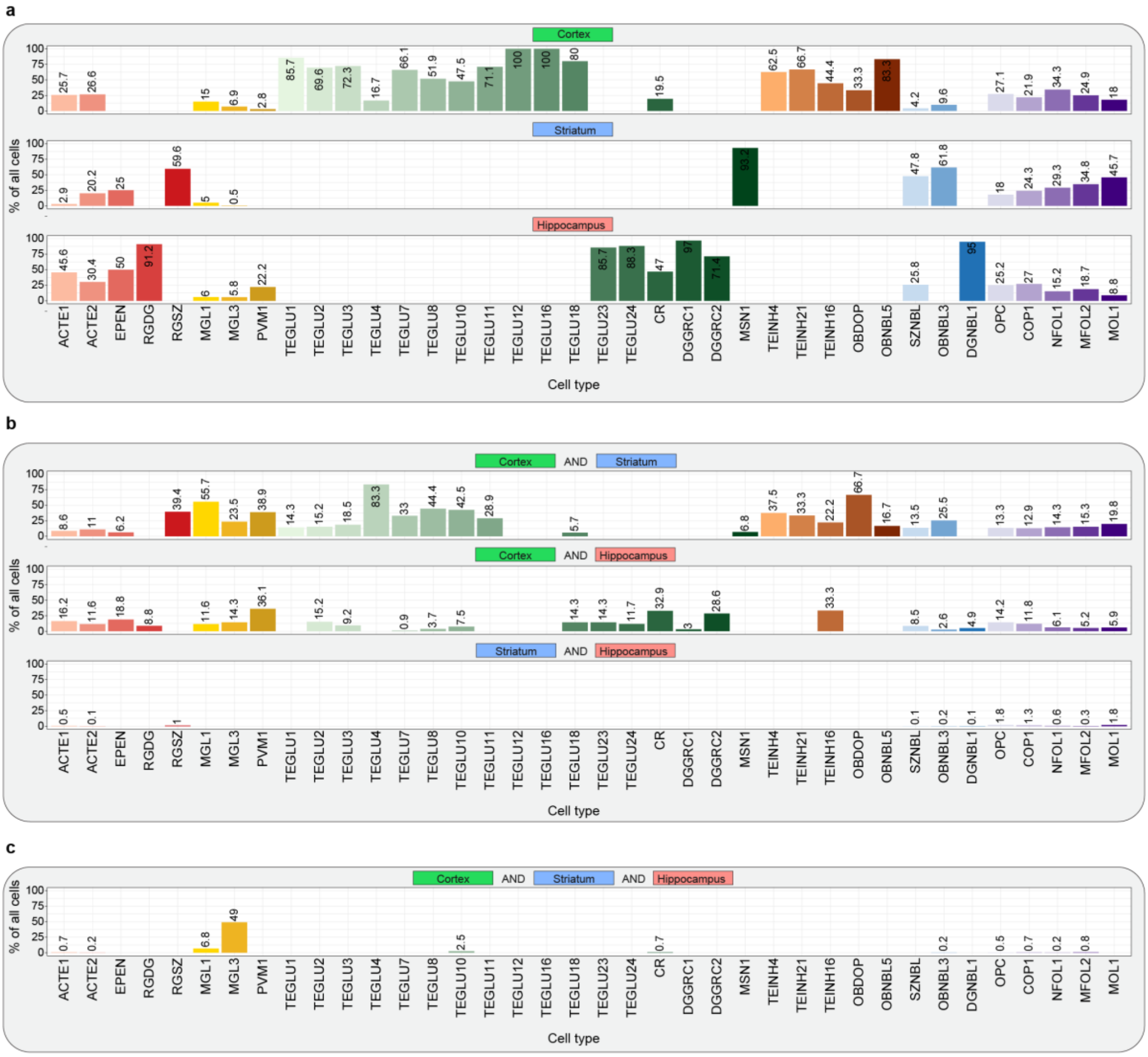

Supplementary Figure 9. Proportions of cells per region for each cell type.

a-c, To assess which cell types were associated with dispersed clones and how often they crossed anatomical boundaries between cortex, striatum and hippocampus, we determined the cell type composition of clones located in a single region (a), two regions (b) or all three regions (c).

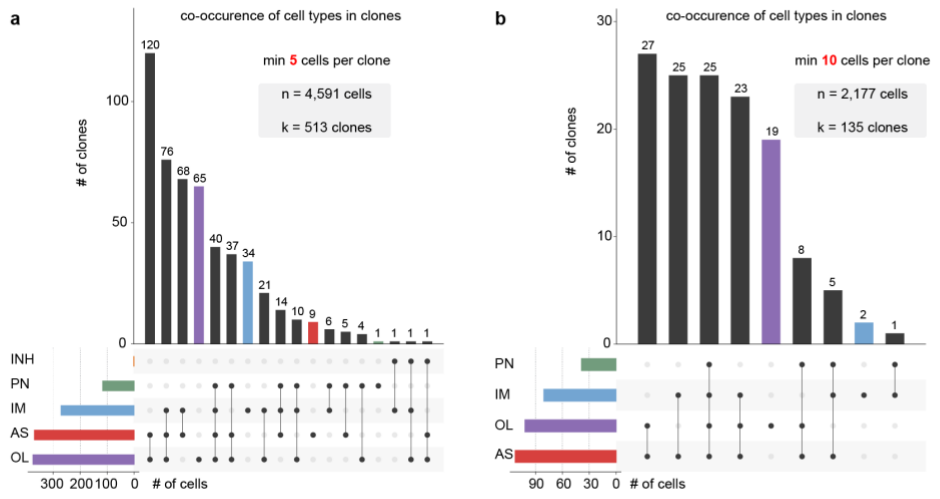

Supplementary Figure 10. Fate distributions of E9.5 neuroectoderm-derived cells.

a, **b**, The number of "minimally uni-potential" clones decreases with increasing clone size. Only clones with neuroectoderm-derived cells containing at least 5 cells per clone (**a**) or at least 10 cells per clone (**b**) are plotted.

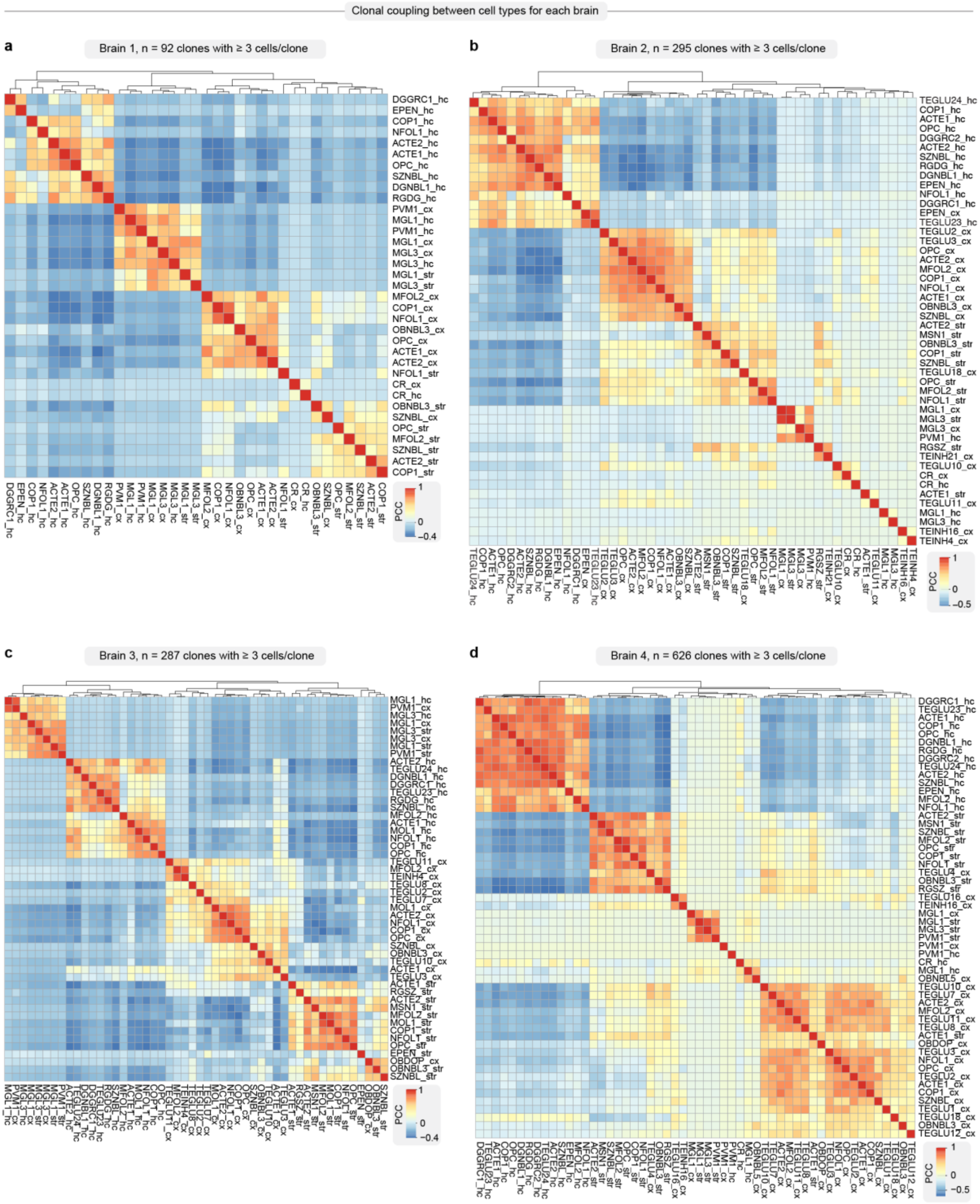

Supplementary Figure 11. Clonal coupling scores for each brain.

a-d, Clonal coupling z-scores defined as the number of shared cloneIDs between all pairs of cell types relative to randomized data were calculated for clonally related cells isolated from brain 1 (**a**), brain 2 (**b**), brain 3 (**c**) and brain 4 (**d**) followed by pairwise correlation of z-scores. Complete-linkage clustering of correlated z-scores revealed structured groups of clonally related cell types as indicated by positive

Pearson correlation coefficient (PCC). Clones containing at least 3 cells per clone were considered for each brain.

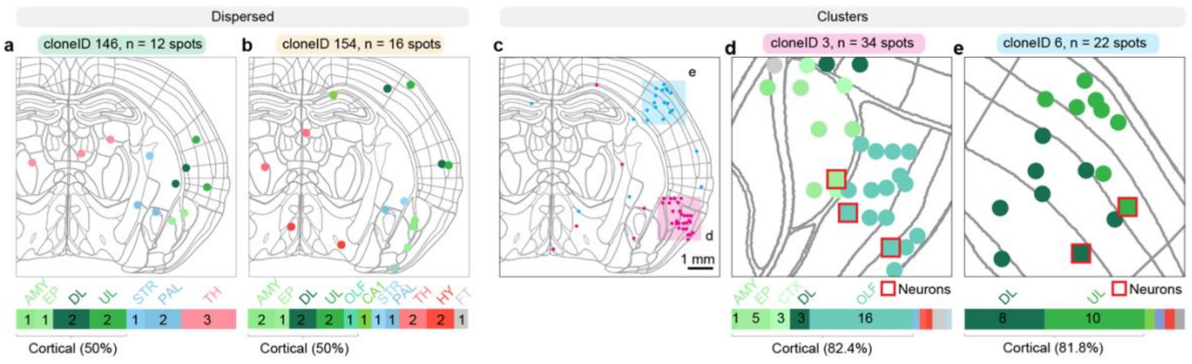

Supplementary Figure 12. Clonal dispersion and clustering using Space-TREX.

Examples of dispersed clones with detailed regional color code of spots. **c-e**, Examples of clustered clones containing color-coded regional information and the cell type "neuron" encoded as red squares.

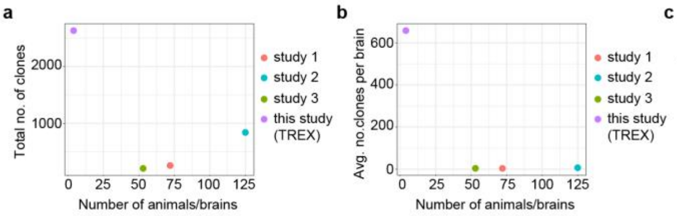

c

	study 1	study 2	study 3	this study (TREX)
**Number of clones**	260	841	210	2 630
**Number of animals/brains**	72	125	53	4
**Average number of clones per brain**	3.61	6.73	3.96	658

Supplementary Figure 13. Number of tracked clones using different methods.

We compared the number of clones detected and animals used between studies that use classical fate mapping methods based on ultra-sparse labeling of progenitors (retroviral tracing, Mosaic Analysis with Double Markers, Cre-induced genetic labeling) and high-density clonal tracing using TREX. **a, b,** Scatter plots showing the total number animals/brains used and the total number of clones detected (a) or the total number animals/brains used and the average number of clones per brain detected (b). **c,** Table summarizing values for all parameters (rows) and studies (columns). The three studies refer to important papers in the field of neurodevelopment published between 2017 and 2020 where the relevant information was accessible: study 1 (Beattie and Hippenmeyer, FEBS Letters, 2017), study 2 (Llorca et al., eLife, 2019), study 3 (Cadwell et al., eLife, 2020).
