# Supplementary material for: Clonal relations in the mouse brain revealed by single-cell and spatial transcriptomics
Source: Nat Neurosci. 2022 Feb 24;25(3):285–94. doi: 10.1038/s41593-022-01011-x (PMC8904259; doi:10.1038/s41593-022-01011-x)
Supplement: Supplementary file 2 — Reporting Summary [file 41593_2022_1011_MOESM2_ESM.pdf]

nature research

Corresponding author(s): Jonas Frisén

Last updated by author(s): Jan 4, 2022

Reporting Summary

Nature Research wishes to improve the reproducibility of the work that we publish. This form provides structure for consistency and transparency in reporting. For further information on Nature Research policies, see our Editorial Policies and the Editorial Policy Checklist.

Statistics

For all statistical analyses, confirm that the following items are present in the figure legend, table legend, main text, or Methods section.

n/a	Confirmed	
☐	☒	The exact sample size (*n*) for each experimental group/condition, given as a discrete number and unit of measurement
☐	☒	A statement on whether measurements were taken from distinct samples or whether the same sample was measured repeatedly
☐	☒	The statistical test(s) used AND whether they are one- or two-sided<br>*Only common tests should be described solely by name; describe more complex techniques in the Methods section.*
☒	☐	A description of all covariates tested
☐	☒	A description of any assumptions or corrections, such as tests of normality and adjustment for multiple comparisons
☐	☒	A full description of the statistical parameters including central tendency (e.g. means) or other basic estimates (e.g. regression coefficient) AND variation (e.g. standard deviation) or associated estimates of uncertainty (e.g. confidence intervals)
☒	☐	For null hypothesis testing, the test statistic (e.g. F, t, r) with confidence intervals, effect sizes, degrees of freedom and P value noted<br>*Give P values as exact values whenever suitable.*
☒	☐	For Bayesian analysis, information on the choice of priors and Markov chain Monte Carlo settings
☒	☐	For hierarchical and complex designs, identification of the appropriate level for tests and full reporting of outcomes
☐	☒	Estimates of effect sizes (e.g. Cohen's *d*, Pearson's *r*), indicating how they were calculated

Our web collection on statistics for biologists contains articles on many of the points above.

Software and code

Policy information about availability of computer code

Data collection	For single cell transcriptomics, brain tissue was dissociated and single cell suspensions were processed using the 10X Genomics Chromium Single Cell Kit Version 2 or Version 3 following the manufacturer's instructions. Spatial transcriptomics was performed using Visium Spatial Gene Expression (10X Genomics) compatible with immunohistochemistry and imaging was done with an epifluorescence microscope (Axio Imager.Z2, Carl Zeiss). DNA sequencing libraries from plasmid DNA or full length cDNA were prepared using custom protocols. All libraries were sequenced using Illumina NovaSeq6000, NextSeq550, or MiSeq. Fluorescent activated cell sorting was performed using a BD Influx equipped with a 140 µm nozzle and a cooling unit.
Data analysis	Raw sequencing data were processed using Cell Ranger v3.0.1 (10X Genomics Chromium) or Space Ranger v.1.0.0 (10X Genomics Visium), respectively. CloneIDs were extracted from output BAM files using a custom Python pipeline published together with this work (https://github.com/frisen-lab/TREX). For clone calling we used the "clone calling function" with a correlation.cutoff of 0.7 (https://github.com/morris-lab/BiddyetalWorkflow/blob/master/scripts/CellTagCloneCalling_Function.R).<br>Data analysis and visualization was done using R v4.1.1 (BiRewire v3.6.0, cowplot v1.1.1, dplyr v1.0.7, EBimage v3.14, eulerr v6.1.1, extraDistr v1.9.1, ggplot2 v3.3.5, magick v2.7.3, magrittr v2.0.1, Matrix v1.4-0, pheatmap v1.0.12, proxy v0.4.26, RColorBrewer v1.1.2, reshape2 v1.4.4, rgl v0.1, Seurat v3, STutility v0.1.0, tidyverse v1.3.1, umap v0.2.7.0, wholebrain v0.1, zeallot 0.1.0) and Python v3 (AmpUMI, Loompy v3.0.6, NumPy v1.19.0, Pandas v1.3.5, Pysam v0.18.0, Tinyalign v0.2-3, xopen v0.1.0) packages.<br>Registration of brain tissue sections to the standardized Allen Mouse Brain Atlas was done using WholeBrain (https://github.com/tractatus/wholebrain). Image segmentation was done using a custom R pipeline published together with this work (https://github.com/ludvigla/TREXSeg).<br>Fluorescent activated cell sorting data were analyzed using FlowJo v10.

For manuscripts utilizing custom algorithms or software that are central to the research but not yet described in published literature, software must be made available to editors and reviewers. We strongly encourage code deposition in a community repository (e.g. GitHub). See the Nature Research guidelines for submitting code & software for further information.

Data

Policy information about availability of data

All manuscripts must include a data availability statement. This statement should provide the following information, where applicable:

- Accession codes, unique identifiers, or web links for publicly available datasets
- A list of figures that have associated raw data
- A description of any restrictions on data availability

Raw data and counts matrices are available at Gene Expression Omnibus (GEO) under accession code GSE153424. All processed single-cell and spatial transcriptomics datasets are available as RDS files using link https://kise-my.sharepoint.com/:f:/g/personal/michael_ratz_ki_se/EndBZ9VI_rRHmHzZxrAwSZQBeE9e4RNmktbuCcHir1a5qQ?e=Ge2Fqm and password 8RMG.xbzH?3v9Ef4

Field-specific reporting

Please select the one below that is the best fit for your research. If you are not sure, read the appropriate sections before making your selection.

☒ Life sciences ☐ Behavioural & social sciences ☐ Ecological, evolutionary & environmental sciences

For a reference copy of the document with all sections, see nature.com/documents/nr-reporting-summary-flat.pdf

Life sciences study design

All studies must disclose on these points even when the disclosure is negative.

Sample size

No statistical method was used to predetermine sample size, but our sample sizes (TREX: five mouse brains, three regions each; Space-TREX: one mouse brain, four control sections and four sections for IHC staining) match typical numbers used in scRNA-seq and Spatial Transcriptomics experiments (Tasic et al 2016; Tasic et al 2018; Zeisel et al 2018; Hochgerner et al 2018; Stahl et al 2016).
For TREX one male and one female EGFP+ animal was randomly selected from two different litters and the control mouse was randomly selected from a third litter. For Space-TREX one EGFP+ mouse was randomly selected from a pool of littermates. For TREX we collected all EGFP+ (barcoded) cells for each brain region to sample the maximum amount of clonally related cells and to have enough cells for each cell type allowing further quantitative analysis such as differential gene expression or lineage coupling.

Data exclusions

No data were excluded.

Replication

The number of replicates is indicated in each experiment:
Fig, 1b: n = 3 brains; Fig. 1c: n = 3 brains; Fig. 1d: n = 5 brains; Fig. 5b: n = 8 sections; Fig. 5b: n = 8 sections; Fig. 5c: n = 4 sections; Extended Data Fig. 1c: n = 4 virus preparations; Extended Data Fig. 2b: n = 3 brains; Extended Data Fig. 2c: n = 3 brains; Extended Data Fig. 2e-g: n = 3 brains; Extended Data Fig. 3b: n = 4 brains; Extended Data Fig. 4d: n = 4 brains; Extended Data Fig. 8h: n = 4 sections; Extended Data Fig. 9b: n = 9 sections. All attempts at replication have been successful.

Randomization

For TREX one male and one female EGFP+ animal was randomly selected from two different litters and the control mouse was randomly selected from a third litter. For Space-TREX one EGFP+ mouse was randomly selected from a pool of littermates. The allocation to experimental groups (barcoded vs. control) could not be randomized, because it was necessary to specifically isolate EGFP+ cells present only in barcoded brains for our approach.

Blinding

Blinding was not applicable for any experiments or data analysis, because control and barcoded samples were of similar age, differentiable based on EGFP fluorescence and our results were based on analysis of clonal barcodes only present in EGFP+ cells.

Reporting for specific materials, systems and methods

We require information from authors about some types of materials, experimental systems and methods used in many studies. Here, indicate whether each material, system or method listed is relevant to your study. If you are not sure if a list item applies to your research, read the appropriate section before selecting a response.

Materials & experimental systems

n/a	Involved in the study	
☐	☒	Antibodies
☒	☐	Eukaryotic cell lines
☒	☐	Palaeontology and archaeology
☐	☒	Animals and other organisms
☒	☐	Human research participants
☒	☐	Clinical data
☒	☐	Dual use research of concern

Methods

n/a	Involved in the study	
☒	☐	ChIP-seq
☐	☒	Flow cytometry
☒	☐	MRI-based neuroimaging

Antibodies

Antibodies used	non-conjugated primary antibodies:<br>EGFP (chicken, 1:2000, Aves Labs, AB_2307313)<br>NeuN (rabbit, 1:500, Atlas Antibodies, AB_10602305)<br>Sox9 (goat, 1:300, R&D Systems, AB_2194160)<br>Sox10 (goat, 1:300, R&D Systems, AB_442208)<br>Iba1 (rabbit, 1:500, Wako, AB_839504)<br><br>conjugated secondary antibodies (all 1:500):<br>donkey anti-chicken conjugated to Alexa Fluor 488 (Jackson Immuno, 703-545-155, AB_2340375)<br>donkey anti-rabbit conjugated to Alexa Fluor 647 (Jackson Immuno, 711-605-152, AB_2492288)<br>donkey anti-goat conjugated to Alexa Fluor 647 (Jackson Immuno, 705-605-147, AB_2340437)<br><br>conjugated primary antibodies:<br>FluoTag®-X4 anti-GFP conjugated to Atto488 (1:200, NanoTag Biotechnologies, N0304)<br>NeuN-Alexa568 (rabbit, 1:400, Abcam, ab207282)<br>Olig2-Alexa647 (rabbit, 1:200, Abcam, ab225100)
Validation	All antibodies are commercially available and tested in immunohistochemical applications in cells and tissue. The antibodies are well described and specific references can be found on the manufacturer's website and in the antibody registry (https://antibodyregistry.org/) using the above given reference numbers.

Animals and other organisms

Policy information about studies involving animals; ARRIVE guidelines recommended for reporting animal research

Laboratory animals	CD-1 mice (1x P11 female; 1x P11 male; 1x P12 male; 1x P12 female; 1x P14 male, 1x P14 female) obtained from Charles River Germany were used for all experiments. Animals were housed in standard housing conditions (ambient temperature of 20-22°C and humidity of 40-60%) with 12:12-hour light:dark cycles with food and water ad libitum. All experimental procedures were approved by the Stockholms Norra Djurförsöksetiska Nämnd.
Wild animals	The study did not involve wild animals.
Field-collected samples	The study did not involve field-collected samples.
Ethics oversight	All experimental procedures were approved by the Stockholms Norra Djurförsöksetiska Nämnd.

Note that full information on the approval of the study protocol must also be provided in the manuscript.

Flow Cytometry

Plots

Confirm that:

☒ The axis labels state the marker and fluorochrome used (e.g. CD4-FITC).

☒ The axis scales are clearly visible. Include numbers along axes only for bottom left plot of group (a 'group' is an analysis of identical markers).

☒ All plots are contour plots with outliers or pseudocolor plots.

☒ A numerical value for number of cells or percentage (with statistics) is provided.

Methodology

Sample preparation	Mice were sacrificed with an overdose of isoflurane, followed by transcardial perfusion with ice cold artificial cerebrospinal fluid (aCSF, in mM: 87 NaCl, 2.5 KCl, 1.25 NaH_2PO_4, 26 $NaHCO_3$, 75 sucrose, 20 glucose, 2 $CaCl_2$, 2 $MgSO_4$). Mice were decapitated, the brain was collected in ice-cold aCSF, 1 mm coronal slices collected using an acrylic brain matrix for mouse (World Precision Instruments) and the regions of interest microdissected under a stereo microscope with a cooled platform. Tissue pieces were dissociated using the Papain dissociation system (Worthington Biochemical) with an enzymatic digestion step of 20-30 min followed by manual trituration using fire polished Pasteur pipettes. Dissociated tissue pieces were filtered through a sterile 30 µm aCSF-equilibrated Filcon strainer (BD Biosciences) into a 15 ml centrifuge tube containing 9 ml of aCSF and 0.5% BSA. The suspension was mixed well, cells were pelleted in a cooled centrifuge at 300 x g for 5 min, supernatant carefully removed, and cells resuspended in 1 ml aCSF containing reconstituted ovomucoid protease inhibitor with bovine serum albumin. A discontinuous density gradient was prepared by carefully overlaying 2 ml undiluted albumin-inhibitor solution with 1 ml of cell suspension followed by centrifugation at 100 x g for 6 minutes at 4°C. The supernatant was carefully removed, the cell pellet resuspended in 1 ml aCSF containing 0.5% BSA and the cell suspension transferred to a round bottom tube (BD Biosciences) for flow cytometry.
Instrument	BD Influx

Software	Data was collected using BD FACS software (Influx) and analyzed using FlowJo v10.
Cell population abundance	Samples were sorted at 1000-1500 events/sec using the 140 um nozzle and a cooling unit with sample temperature of 4°C achieving >90% purity by FACS analysis.
Gating strategy	Barcoded cells isolated from experimental animals express EGFP and were defined by comparison to non-lentivirus transduced control samples.

☒ Tick this box to confirm that a figure exemplifying the gating strategy is provided in the Supplementary Information.
